# Speciation in little: the role of range and body size in the diversification of Malagasy mantellid frogs

**DOI:** 10.1186/1471-2148-11-217

**Published:** 2011-07-21

**Authors:** Katharina C Wollenberg, David R Vieites, Frank Glaw, Miguel Vences

**Affiliations:** 1 Department of Organismic and Evolutionary Biology & Museum of Comparative Zoology, Harvard University, 26 Oxford St., Cambridge, MA 02134, USA; 2 Museo Nacional de Ciencias Naturales, CSIC, C/José Gutierrez Abascal 2, Madrid 28006, Spain; 3 Zoologische Staatssammlung München, Münchhausenstraße 21 ,81247 München, Germany; 4 Zoological Institute, Division of Evolutionary Biology, Technical University of Braunschweig, Mendelssohnstr 4, 38106 Braunschweig, Germany

## Abstract

**Background:**

The rate and mode of lineage diversification might be shaped by clade-specific traits. In Madagascar, many groups of organisms are characterized by tiny distribution ranges and small body sizes, and this high degree of microendemism and miniaturization parallels a high species diversity in some of these groups. We here investigate the geographic patterns characterizing the radiation of the frog family Mantellidae that is virtually endemic to Madagascar. We integrate a newly reconstructed near-complete species-level timetree of the Mantellidae with georeferenced distribution records and maximum male body size data to infer the influence of these life-history traits on each other and on mantellid diversification.

**Results:**

We reconstructed a molecular phylogeny based on nuclear and mitochondrial DNA for 257 species and candidate species of the mantellid frog radiation. Based on this phylogeny we identified 53 well-supported pairs of sister species that we used for phylogenetic comparative analyses, along with whole tree-based phylogenetic comparative methods. Sister species within the Mantellidae diverged at 0.2-14.4 million years ago and more recently diverged sister species had geographical range centroids more proximate to each other, independently of their current sympatric or allopatric occurrence. The largest number of sister species pairs had non-overlapping ranges, but several examples of young microendemic sister species occurring in full sympatry suggest the possibility of non-allopatric speciation. Range sizes of species included in the sister species comparisons increased with evolutionary age, as did range size differences between sister species, which rejects peripatric speciation. For the majority of mantellid sister species and the whole mantellid radiation, range and body sizes were associated with each other and small body sizes were linked to higher mitochondrial nucleotide substitution rates and higher clade diversity. In contrast, small range sizes were unexpectedly associated with a slow-down of mitochondrial substitution rates.

**Conclusions:**

Based on these results we define a testable hypothesis under which small body sizes result in limited dispersal capabilities and low physiological tolerances, causing smaller and more strongly fragmented ranges. This can be thought to facilitate reproductive isolation and thus favor speciation. Contrary to the expectation of the faster speciation of such microendemic phenotype species, we only found small body sizes of mantellid frogs to be linked to higher diversification and substitution rates, but not small range sizes. A joint analysis of various species-rich regional anuran radiations might provide enough species with all combinations of range and body sizes for a more conclusive test of this hypothesis.

## Background

Inferring the processes generating large-scale patterns of biodiversity, especially those shaping adaptive radiations, are fascinating areas of past and current biological research [e.g., [1-3]]. Current debates on diversification focus on speciation modes in the temporal dimension (gradual vs. instantaneous speciation, e.g. through hybrid speciation), the spatial dimension (allopatric, including dichopatric and peripatric, versus parapatric and sympatric speciation), and increasingly on the general mechanisms driving divergence (ecological adaptation, sexual selection, or non-adaptive factors such as genetic drift [4,5]). Life history traits have equally long been discussed as drivers of speciation. Changes in these traits might act as key innovations, promoting ecological opportunity for the emergence of adaptive radiations in the absence of habitat changes [[6-8]; reviewed in [2,3]].

Body size of animal species is such a life history trait that has numerous ecological consequences [9]. For instance, in many taxa body size seems to determine the size of distribution ranges, with smaller taxa having more restricted ranges [10]. This might, at least in some taxa, be simply related to lower dispersal capabilities of smaller species; for instance, a phylogenetically independent positive correlation between home range and body size has been found in ferungulate mammals [11], a result later refined using optimization methods [12].

Adaptive radiation likely evolves in stages [13], with body size being one of the first proposed axes of morphological change leading to new ecological opportunity [[14] as reviewed in [3]]. Body size frequency distributions of animals are generally right-skewed, i.e., most species are generally small [15-17], as for example in 99% of the world's major lizard groups [18] and often the right-skewed shape is kept after log-transformation. In lizards, often there is a strong negative correlation between body size within families and species richness: groups containing mainly small species are more species rich [18]. Similar unimodal body size distributions have been found in other poikilotherm vertebrates (frogs, deep-sea and freshwater fishes), with right-skewedness of the distributions increasing towards the equator [19]. Other researchers found that large radiations of species tend to be small bodied [20], which is in agreement with the hypothesis that the number of ecological niches is potentially greater for small-bodied taxa (although this pattern was not statistically significant), which suggests a correlation between small body size and species richness.

Despite the intuitive nature of the hypothesis that small organisms should have elevated net rates of diversification, an influence of body size on clade richness has been refuted on several taxonomic levels [17]. In a recent study on toads of the cosmopolitan family Bufonidae, Van Bocxlaer and colleagues [21] found that speciose clades were composed mainly of large species with large range sizes. They used a combination of various traits to define an optimal expansion phenotype (OEP) which they invoked to explain the diversification and success of these amphibians. In fact, much of the evolutionary success of bufonids is related to the fact that their radiation was intimately connected to colonization of vast new areas, i.e., continents on which these toads were previously absent [21]. This pattern is opposite than would be expected from a classical explanation for adaptive radiation, where small ranges and specialization by occupation of specific ecological niches are thought to promote diversification within a given area [3].

To test correlation among traits (like body and range size) or influences of such traits on diversification rates, phylogenetic comparative methods (PCM) are applied, ensuring phylogenetic independence of data [22]. Usually, PCM rely on ancestral character state reconstruction along a phylogenetic tree [e.g., [23]]. A generally more robust alternative is the direct comparison of sister species, with each pair of sister species being a phylogenetically independent data point (tip contrasts), but in most data sets there are not sufficient sister species pairs for adequate statistical analysis [22].

Frogs of the family Mantellidae are a highly diverse clade restricted to Madagascar and the Comoroan island of Mayotte, with 100% species-level endemism and over 250 species and candidate species [24]. A large number of mantellid species are small sized, i.e., below 30 mm snout-vent length (SVL), and many are microendemic, i.e., restricted to a very small geographic area. Mantellids have evolved a variety of ecological adaptations, including arboreal, semiaquatic and fully terrestrial species. Given that they diversified within a single geographic setting, these frogs can serve as an excellent model group to test mechanisms of diversification and correlates of diversity, and their large species diversity allows to base PCM calculations on pairs of sister species additional to tree-based methods. Because most amphibian lineages at the genus or family level are restricted to major biogeographic areas [25], and show a strong pattern of regional diversification similar to the mantellids [e.g., [26-28]], we assume that observations on these frogs in Madagascar are more representative for overall amphibian diversification patterns than are bufonid toads with their high dispersal capacity.

In this paper, we reconstruct a near-complete species-level phylogeny of the Mantellidae from nuclear and mitochondrial markers (257 nominal and candidate species). We integrate this phylogeny with 1371 geo-referenced GIS records for 1371 species-locality records [29] and with bioclimatic information. Based on this phylogeny we identify well-supported pairs of sister species. These are then used in sister species comparisons, along with whole-tree based PCM, to identify geographical patterns of speciation, to test the influence of body size on range size, and the combined effect of these two traits on diversification and molecular rates of evolution. We hypothesize that, equivalent to the OEP (Optimal expansion phenotype) characterized by large body and range sizes and observed in successful colonizing lineages in the Bufonidae, the majority of Malagasy mantellid frogs might exhibit a microendemic phenotype (MEP) with small range sizes as a result of their small body size. We also develop the hypothesis that this MEP may have influenced rapid diversification in the group, making them one of the most speciose amphibian radiations to date [30].

## Results

### Molecular phylogeny of the Mantellidae

As the backbone of our species-level phylogeny, we used a reduced-taxa phylogeny recovered for 46 mantellid species of all genera, subgenera and species groups, based on 3760 base pairs of six mitochondrial and nuclear genes. The intrageneric relationships in this mantellid phylogeny were well resolved and highly supported in the Bayesian analysis, with a similar topology recovered by Maximum Parsimony (MP) and Maximum Likelihood (ML) analyses (Additional File 1, Figure S1). All mantellid genera were monophyletic with MP/ML bootstrap support values >95, and Bayesian posterior probabilities >99, with the exception of *Mantidactylus *and *Blommersia*, whose ML bootstrap values were 93%.

The Bayesian analysis of 257 species and confirmed candidate species in the all-taxa data set, including 1772 bp from three mitochondrial genes and using the preferred partitioning strategy (maximal partitioning strategy with the 16S fragment and each of the codon positions for cob and cox1 as separate partitions), yielded a well-supported phylogenetic tree, with high posterior probability values on the levels of species groups (Additional file 1, Figure S2). Most of the subgenera in the Mantellidae [31], especially of *Mantidactylus *and *Gephyromantis*, were found to be monophyletic. Basal relationships among genera in this analysis were constrained on the basis of the previous reduced-taxa topology.

**Figure 1:**
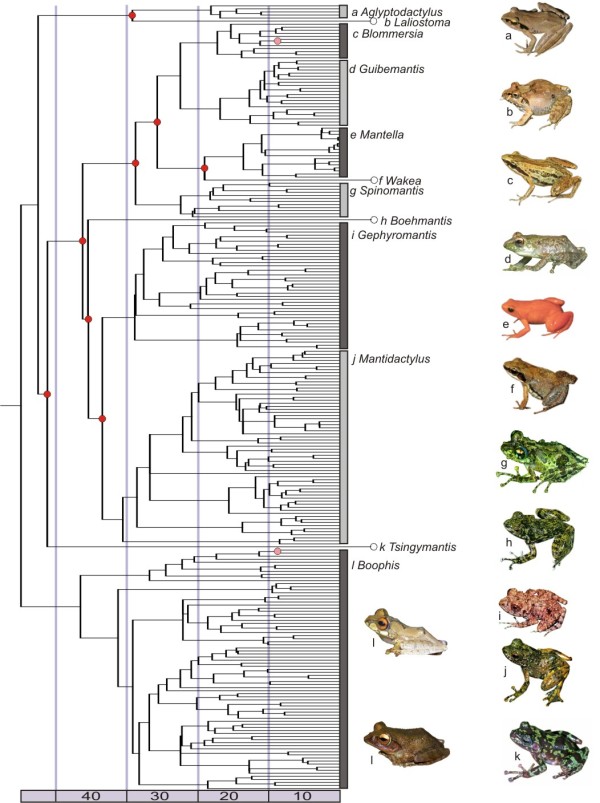
**Timetree of mantellid frogs**. Time-calibrated Bayesian phylogeny obtained with Pathd8 for 257 mantellid species. Circles indicate primary (light red) and secondary (dark red) calibration points. Light and dark bars delimit genera. Purple bar shows estimated age of clades in Ma.

The 50% majority-rule consensus tree obtained from the all-taxa Bayesian analysis was converted into an ultrametric timetree using the software Pathd8 (Figure 1), based on a combination of primary and secondary calibrations (see Materials and Methods). Alternative runs including either only primary or only secondary calibrations recovered similar node ages (not shown). We thus used the combined analysis with all calibrations to obtain evolutionary ages for mantellid sister species, and used these age data in further analyses.

Among the 257 mantellid species in the phylogeny, we identified 53 sister species pairs (19.9% of all species) with high Bayesian posterior probabilities (>95) and within subclades in which we consider taxonomy and distribution ranges to be sufficiently understood. The majority of these (33 = 63.7%) showed no range overlap (allopatric sister species). 20 species pairs (36.4%) showed partial or full overlap of their distribution ranges (sympatric sister species). In 12 of these sympatric pairs we ascertained syntopic occurrence (with distances of 1 km or less between specimens of the two species) in the field, and in two further pairs we ascertained occurrence in the same area, yet at different altitudes. Correlation between evolutionary age and range (Age-range correlation) of sister species pairs was not significant (not shown).

Range overlap among sister species pairs was log-normally distributed, with a mean percentage of range overlap of 13.8%. This value is significantly higher than the mean overlap among all possible combinations of non-sister species in mantellids (8.7%), as indicated by a Sign test (Z = 3.88, p < 0.001). Mean range overlap of the sympatric sister species is generally high (54.2%), with a significantly higher median than all possible combinations of mantellid species when sister species were excluded.

The initial diversification of mantellids was estimated at 44 mya from our time-calibrated phylogeny. The 53 pairs of mantellid sister species were remarkably old, with evolutionary ages ranging from 0.2 mya to 14.4 mya with an arithmetic mean of 7.3 mya, and with node ages following a normal distribution (as proven by non-significant D-statistics of one-sample Kolmogorov-Smirnov test D = 0.09, not shown).

### Spatial characteristics of diversification

**Figure 2:**
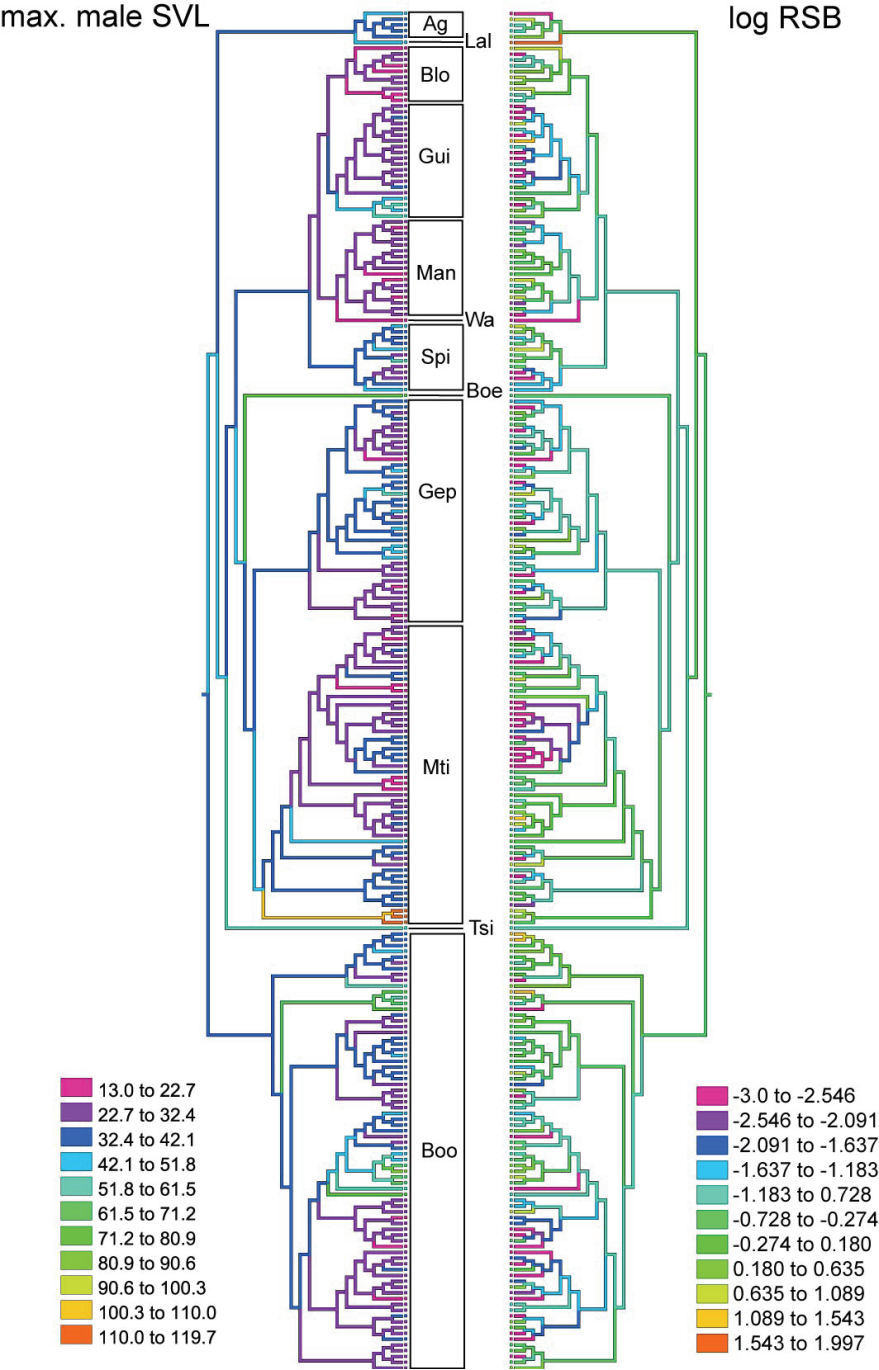
**Maximum Parsimony character tracing of SVL and log RSB**. Visualisation of the distribution of SVL and range size (displayed as logarithm of estimate RSB) over the mantellid species tree created by Maximum Parsimony character tracing in MESQUITE [78] (using the ultrametric topology from Figure 1). Genera abbreviations: Ag - *Aglyptodactylus*, Lal - *Laliostoma*, Blo - *Blommersia*, Gui - *Guibemantis*, Man - *Mantella*, Wa - *Wakea*, Spi - *Spinomantis*, Boe - *Boehmantis*, Gep - *Gephyromantis*, Mti - *Mantidactylus*, Tsi - *Tsingymantis*, Boo - *Boophis*.

**Figure 3:**
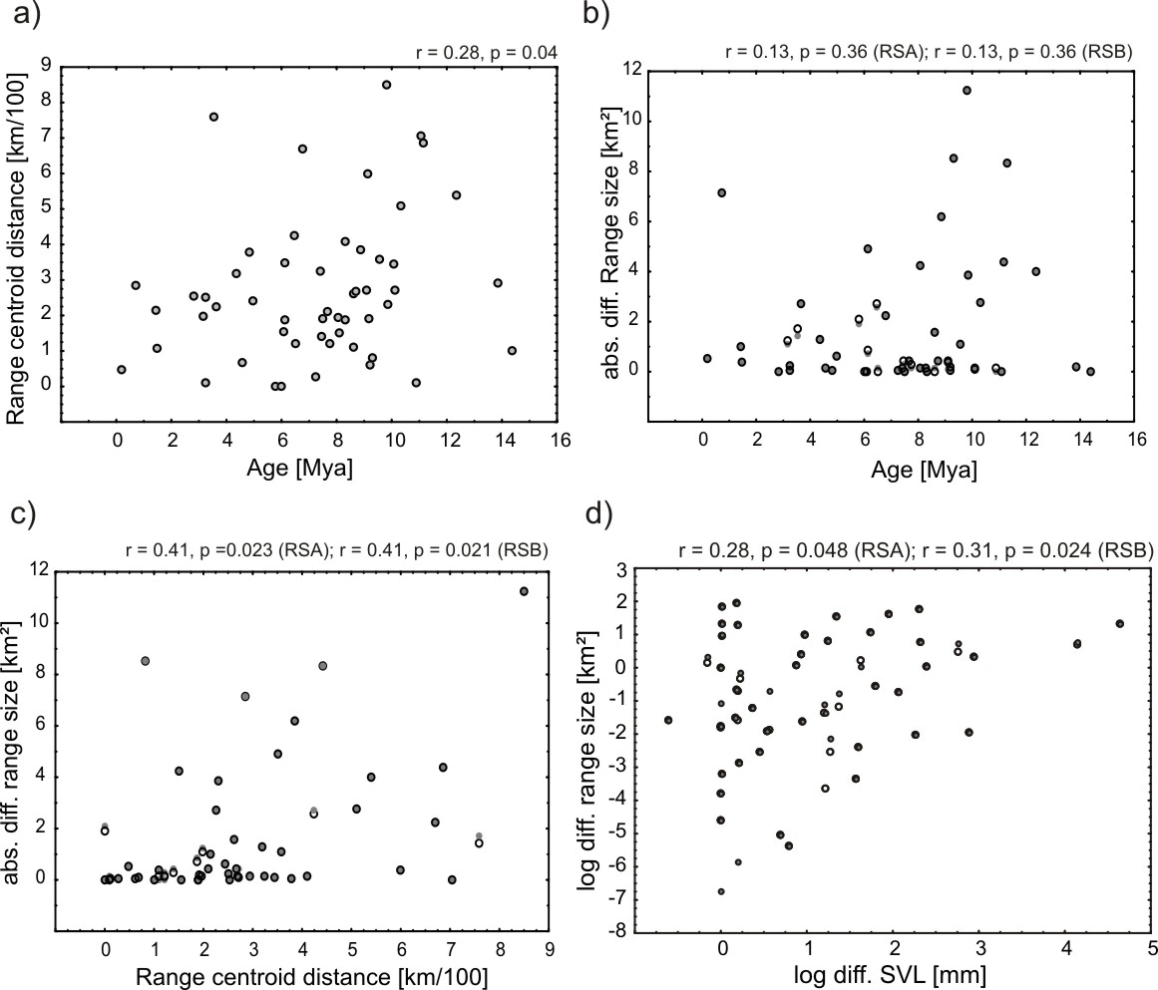
**Relationships between range size, range centroid distance, age and body size in mantellid frogs**. Scatterplots of range and body size correlations for sister species contrasts. Because we used two different range size estimates, we overlaid the plots for these (RSA and RSB). RSA is shown as large open dots and RSB is shown as small filled dots. Datapoints congruent among the two estimates are consequently depicted as large filled dots. a - Range centroid distance increases with age of ss pair. b- Absolute differences in range size increase with age of ss pair. c - range centroid distance increases with range size contrast in sister species. d- log range size tip contrasts of mantellid sister species are correlated with log body size tip contrasts.

Most mantellids had small body sizes and small to very small range sizes (Additional file 1, Figure S3, Figure 2), and this was true also for the subset of species included in the tip contrast comparisons (pairs of sister species). We found a positive correlation between evolutionary age and centroid distance of ranges of pairs of sister species (Figure 3a), which indicates lineage diversification in close spatial proximity.

Patterns related to range size were separately analyzed for two range size estimates (RSA and RSB) that differed in the extent of buffer zones assigned to single-locality species (see Materials and Methods). For the 53 pairs of sister species included in the analysis we found a trend of increasing absolute range size with evolutionary age (r = 0.173, p = 0.073 for RSA; r = 0.175 p = 0.07 for RSB, not shown). All species with most recent splits from their sister species (with one exception, *Mantella ebenaui*) had small range sizes and range size differences (Figure 3b), with larger range sizes and range size differences between sister species being present only in older species (pairs). Under peripatric speciation, a triangular or no correlation among evolutionary age and range size (and consequently sister species range size contrasts) is expected; we therefore performed quantile regression. Quantile regression showed that the 90% quantile slope for absolute range size was positive (0.557), a pattern that is not expected under peripatric speciation where we expected the slope to be negative or zero (see Discussion below). Regression of the 90% quantile found the slopes for absolute differences in RSA (0.656) and RSB (0.656) to be positive. Congruent with these results, the absolute differences in RSA and RSB also increased with centroid distance (Figure 3c).

### Body size as a predictor for range size in the Mantellidae

Range and body sizes for all mantellid species were highly positively correlated (not shown). To ensure phylogenetic independence of these results, standardized independent contrasts for body size and range size estimates were subsequently computed for pairs of sister species [22]. Factorial regression through the origin found contrasts in SVL per mantellid sister species pair to significantly predict tip contrasts of RSA and RSB (Table 1). Sister species with small contrasts of SVL had small range size contrasts (shown as logarithms in Figure 3d, correlation after exclusion of two outlier species pairs). We used the software CoEvol to assess the coupled evolution of SVL and RSA/RSB in a probabilistic framework [32]. Partial regression revealed a significantly positive effect of SVL on both estimates of range size, although the correlation was not particularly strong (Table 2). Multiple regressions performed with each range size estimate and substitution rate did not alter the positive direction of the correlation, however, the posterior probabilities remained only significant for the smaller range size estimate (RSB), but not for RSA (Additional file 1, Table S8).

**Table 1:** Influence of body size on range size in pairs of mantellid sister species

	Multiple R^2^	SS Model	MS Model	SS Residual	MS Residual	F	p
RSA tip contrast	0.265	1102.316	1102.316	3059.713	57.730	19.094	**0.000058**
RSB tip contrast	0.269	1128.238	1128.238	3057.452	57.6877	19.558	**0.000049**

Univariate regression through the origin results to determine the effect of standardized tip contrasts in SVL on range size estimators using large (RSA) and small (RSB) buffer zones for one- or two locality species for mantellid sister species pairs. Significant p-values in bold.

**Table 2:** Phylogenetically independent effects of body and range size on each other and on substitution rate

		Ds	SVL	RSA (0.0158)	RSB (0.001)
Ds	cov	0.314	-0.0308	0.253	0.467
	r^2^	1	0.0246	0.0148	0.0244
	pp	--	0.026	0.87	0.94
SVL	cov		0.124	0.16	0.33
	r^2^		1	0.0152	0.0311
	pp		--	0.96	1
RSA	cov			13.5	14.4
	r^2^			1	0.537
	pp			--	1
RSB	cov				28.3
	r^2^				1
	pp				--

Matrix of covariances, correlation coefficients, and posterior probabilities for maximal male SVL, range size estimates and synonymous substitution rate Ds obtained with CoEvol. Negative covariances indicate negative correlations, posterior probabilities close to zero indicate significant negative correlation, close to 100 indicate significant positive correlations. 1213 generations were sampled and burnin was set = 100 after visual inspection of the trace file as showing stability in estimated parameters.

### The role of body size and range size influencing mantellid diversification

To understand whether body size and range size affect the rate and mode of diversification in the Mantellidae, we first tested whether geographical proximity of ranges and similarities of bioclimatic envelopes of sister species are related to their range and body sizes. A factorial regression analysis revealed standardized contrasts in range size (both RSA and RSB) and body size to be functions of spatial characteristics: microendemic and miniaturized sister species have more proximate ranges and climatically more similar niches than widely distributed or larger species (Table 3). Univariate results for the single predictors and their interaction terms can be found in Additional file 1, Table S7.

**Table 3:** Effects of evolutionary age and spatial distance on range and body size

	Multiple R^2^	SS Model	MS Model	SS Residual	MS Residual	F	p
SVL tip contrast	0.621	4743.239	677.606	2900.391	67.451	10.046	**<0.0001**
RSA tip contrast	0.633	2632.051	376.007	1527.497	35.524	10.585	**<0.0001**
RSB tip contrast	0.636	2659.325	379.903	1523.884	35.439	10.719	**<0.0001**

Results of factorial regressions through the origin to determine the effect of evolutionary age, of geographic distance and bioclimatic distance, and their interaction terms on body size and range size contrasts using large (RSA) and small (RSB) buffer zones for one- or two locality species in mantellid sister species pairs.

**Figure 4:**
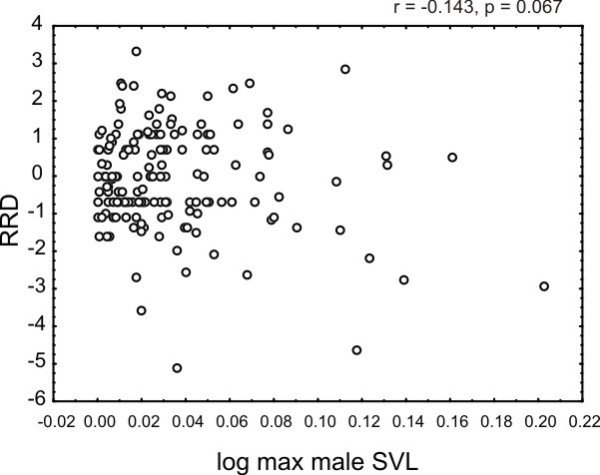
**Correlation between SVL and clade richness contrast**. Negative correlation between RRD (species richness contrast) and logSVL as inferred with MacroCAIC, although this was not significant.

We used the software MacroCAIC as a whole-tree based PCM to test whether mantellid lineages with smaller SVL are more speciation-prone than lineages showing larger SVL [33]). Both lineage richness and SVL contrasts were normally distributed (as assessed with Kolmogorov-Smirnov tests in STATISTICA, results not shown), so we could perform standard regression through the origin [11]. The null hypothesis of small SVL not influencing clade diversification rate could not be rejected with this test (F = 2.78, R^2 ^= 0.0166, p = 0.0972). However, the negative slope indicates that clades exhibiting smaller SVL have a trend of being more species-rich (r = -0.143, p = 0.067; Figure 4). Effects of RSA and RSB on clade diversification were, however not detectable at all with this test (p = 0.37 for RSA and p = 0.55 for RSB, respectively, not shown). A strong influence of small body and range sizes not on the number of lineages but on the mitochondrial substitution rate itself was detected by phylogenetic regression in CoEvol [32] (Table 2). The approximated synonymous substitution rate of the all-taxa dataset was, given the ultrametric phylogeny, significantly negatively correlated with SVL; small body size turned out to be associated with high rates of molecular substitution. This result remained unchanged if multiple regressions including RSA and RSB were performed (Additional file 1, Table S8). In contrast, the two range size estimates were positively correlated with the substitution rate (if only very weakly), which means that in extant species that are both small and have small range sizes (exhibiting a MEP), substitution rate would experience a relative slow-down compared to lineages that have small body sizes but large range sizes. These results were supported in a multiple regression by high posterior probabilities for RSB, but not for the larger minimal range size estimate RSA (Additional file 1, Table S8).

## Discussion

### Characteristics of the mantellid radiation

Most mantellid sister species lineages diversified between 7 and 8 mya. These generally old ages (upper Miocene) indicate that Pleistocene speciation mechanisms as they have been proposed for lemurs [34] cannot be invoked to explain the bulk of mantellid speciation, and instead unveil an older speciation timing similar to that detected in a diverse range of Neotropical taxa [26,35] and in other Malagasy animals [36]. About 64% of mantellid sister species did not show range overlap, a percentage that is consistent with an average value of 72% allopatric species estimated for other animal clades [23,37-40]. Mantellid frogs show a right-skewed body size species richness pattern, contrary to what has been observed in Malagasy mammals [41].

### Speciation in close spatial proximity and rejection of peripatric speciation

Although methods that attempt to quantify the frequency of sympatric versus allopatric speciation exist, e.g., age-range overlap correlation [23,37,42,43], these have been widely criticized when applied to fast-dispersing groups of taxa due to the high probability of post-speciation range shifts and because their results usually do not differ from null models [1]. Most amphibians, and many mantellids in particular, are probably no fast dispersers, so conclusions drawn from age-range overlap correlation in this group might thus be less compromised than in other groups. We found sympatric and allopatric sister species to be present in all major mantellid lineages and to span similar evolutionary age ranges. Youngest pairs of sister species were both distributed in sympatry and in allopatry, which resulted in the absence of a correlation between evolutionary age and range overlap. Such absence of age-range overlap correlation can either be caused by post-speciation range shifts, or by a mix of different geographical modes of speciation [1]. In fact, our results bear visual similarities to the modeled data under multiple speciation modes [43]. Alternatively, Pleistocene climate oscillations that postdate the time of maximal lineage diversification in mantellid frogs can be thought of having triggered post-speciation range shifts [23]. Besides the many microendemic mantellids, also widespread species do exist in this family [29], so we cannot conclusively distinguish between the hypotheses of (i) a mix of speciation modes, or (ii) absence of a clear pattern due to range shifts.

However, a clear conclusion derived from our data is that lineage diversification in mantellid frogs typically happens in close spatial proximity, since youngest mantellid sister species pairs were also the most geographically proximate and spatial distance gradually increased with evolutionary age. Under sympatric speciation, the geographical proximity is obvious, and under allopatric speciation, the initial geographic separation of populations of the ancestral species is likely to be small in non-vagile animals - except for cases of overseas dispersal [44] or speciation of relict populations in isolated habitats.

In fact, in several species pairs with almost fully overlapping ranges, the currently available evidence would favor sympatric or parapatric species formation, and we flag them for future detailed study. Some of these are old species, such as *Gephyromantis azzurrae *and *G. corvus *(estimated divergence 10.9 ma), both of which are endemic to a very small range in the Isalo sandstone massif in south-western Madagascar, or *Spinomantis bertini *and *S*. sp. 6 (estimated divergence 8.6 ma) which occupy different altitudinal bands on the Andohahela massif in the south-east. *Boophis majori *and *B*. sp. 35 (estimated divergence 7.2 ma) occur in syntopy in Ranomafana National Park in the southern central east, and differ strongly by advertisement calls and tadpole morphology [24]. And one of the youngest mantellid sister species pairs, *Gephyromantis eiselti *and *G. thelenae *(estimated divergence 3.2 ma), two species with endotrophic tadpole development, even form mixed choruses at some sites near Andasibe in the northern central east, with pronounced differences in advertisement calls [45,46]. Despite these examples, the high proportion of allopatric pairs of sister species in the Mantellidae as apparent from our analysis suggests that allopatric modes of species formation probably have played a predominant role in the diversification of these frogs.

Range sizes of species included in the sister species comparisons increased with evolutionary age, as did range size differences between sister species. This is not trivial as it firmly rejects peripatric speciation [47] as predominant speciation mode for mantellid frogs (under which we would assume range asymmetry in youngest sister species to be high [48]). Speciation in mantellids mostly resulted in microendemic sister species (with proximate centroids), a pattern not expected under predominance of species formation based on peripheral, allopatric isolation of small subpopulations as in peripatric speciation. A probable explanation for some mantellid frogs being widespread is that their ecological tolerance is wider, which would make the interruption of gene flow to peripheral populations by ecogeographic barriers less likely. In contrast, narrow-range species are likely habitat specialists, which increases the probability of allopatric populations to become isolated, e.g. by habitat changes through climatic shifts. Small adaptive changes in such a population can already confer a significant shift in habitat preference, thus leading to genetic isolation in parapatry or sympatry.

### A role for a microendemic phenotype in lineage diversification?

As a first hypothesis we tested whether our data support the existence of a microendemic phenotype (MEP), i.e., whether frogs of small body size also have particularly small range sizes. If this pattern is phylogenetically independent, we can conclude evolutionary convergence of body size and small range sizes forming a distinct MEP. Using robust tip-based PCM based on a high number of mantellid sister species pairs we found SVL to be a significant predictor of range size. The correlation between range size and SVL was positive, supporting that the combination of small SVL and small range size defines a MEP. Whole-tree based methods support this pattern: CoEvol found a phylogenetically independent, significant positive correlation between SVL and RSB, reflecting the fact that most mantellids are both small and have small range sizes, and hinting at replicated evolution of this phenotype within the mantellid radiation. As a second hypothesis, we posit that lineages exhibiting MEP will be less dispersal-prone and therefore will be less able to maintain gene flow among populations (due to small body size equaling low dispersal capabilities), leading to increased rates of speciation. If the MEP drives diversification in the Mantellidae, clades exhibiting MEP are thus expected to be more species rich and to inhabit ranges in close spatial proximity. Using PCM based on mantellid sister species pairs we found small body and range size contrasts to be prevalent in proximate and young sister species, thus supporting the hypothesis. The application of tree-based PCM revealed more ambiguous results to this question. MacroCAIC found the expected negative regression slope between diversification rate and SVL, but the regression through the origin could not reject the null hypothesis of no effect of SVL on diversification rate (albeit with a low rejection error probability of 0.097). The effect size of the correlation, however, almost reached significance (0.067), but no effects of range size on clade diversification could be detected. While these analyses using clade diversity remained ambiguous, we found effects of both SVL and range size (RSB) on the synonymous mitochondrial substitution rate. While small SVL predicted a high rate of substitution, range size in contrast was positively associated with the substitution rate: larger-range lineages show higher rates of substitution. Mitochondrial substitution rates have been found to be positively correlated with speciation rates and contemporary species richness before [49]. If we assume a similar relationship in the Mantellidae, the positive correlation between range sizes and substitution rate would contradict the hypothesis that MEP (lineages with both small range and body sizes) promotes diversification. Small-bodied species that also have small range sizes (the majority of the extant mantellid species) would experience a net reduction in substitution rate, and presumably in diversification rate, as compared to lineages with small SVL but less microendemic distributions. A slow-down of speciation rate is, however, implied in the definition of adaptive radiations - after environmental niches are occupied in the later stages of adaptive radiation, lineages cannot continue to diversify as rapidly [7,50,51]. A similar slow down of diversification rate with decreasing range size has been predicted [50] under the scenario that small range sizes are indicative of strong ecological competition from close relatives in adaptive radiations. However, a stronger rate slow-down in a bird dataset has been found be related to larger ranges [50], contrary to the expectation. In general, the association of rates of clade diversification and molecular substitution clearly require further study. Another factor possibly related to the pattern observed is population density: in small frogs, a viable population could be established in smaller ranges than in large frogs [51], potentially driving the correlated evolution of microendemism. However, the lack of data on population densities for mantellid frogs (and most other tropical amphibians) does not permit testing this hypothesis at present.

From our results, we conclude that our data support an association of small range and body size and thus the existence of a MEP. We furthermore find indications that not the MEP, but instead small body sizes might be crucial in promoting lineage diversification. Extant mantellids show a high frequency of MEP lineages, but instead of having a combined high diversification rate, these lineages might experience a net slow down of diversification and substitution rates, as expected in the late stages of adaptive radiation. We can back up the results derived from tree-based PCM (that might be sensitive to errors in phylogenetic reconstruction) with tip-based PCM using a high number of sister species pairs. Although we implicitly assume that the sister lineages are characterized by the extant state since they diverged at the nodes in tip-based PCM, these can complement tree-based PCM in near-complete phylogenies.

## Conclusions

The MEP hypothesis complements the optimal expansion phenotype (OEP) hypothesis [21], residing at the other end of a phenotypic continuum. The phenotypes at both extremes of this continuum promote fast successful speciation, but are based on different mechanisms. The OEP promotes speciation by providing the ability to colonize vast ranges that create ecological opportunity [52] while the MEP promotes rapid speciation on a smaller spatial scale because of its association with low dispersal capacity, and restriction of gene flow by making geographic barriers relatively larger [53]. The OEP contradicts the expectations from a classical adaptive radiation [21], where small ranges and specialization by occupation of specific ecological niches are thought to promote diversification within a given area [reviewed in [1]]. Our results provide evidence for the existence of an MEP in the Mantellidae, but contrary to the expectation of the faster diversification in microendemic phenotype species, the majority of extant mantellids (MEP species) that show small body sizes combined with small range sizes can be thought of experiencing a relative slowed-down rates of speciation compared to small but not microendemic species. A joint analysis of various species-rich regional anuran radiations might provide enough species with all combinations of range and body sizes for a more conclusive test of the influence of body and range size on the diversification rate in amphibians.

## Methods

### Time-calibrated molecular phylogeny

We compiled a near-complete set of samples for species and candidate species of the Mantellidae. Only the described mantellid species *Spinomantis brunae, S. nussbaumi*, and *S. tavaratra *are missing ("all-taxa data set"). We are aware that new mantellid species will continue being discovered in the future [24]. Along with other thorough molecular phylogenetic studies of species-rich tropical amphibian radiations [e.g., [26,27]] we are confident to have assembled the most complete such data set to date. To define candidate species, we followed an integrated approach that combined genetic divergence with bioacoustic and morphological characters [24,48,54,55]. For most mantellid species, genetic data was available for more than one population and individual, and these as well as bioacoustic and morphological data support a status as valid species for the undescribed species included in this study [24].

We compiled two sets of DNA sequences: (i) a combined mitochondrial/nuclear gene data set to reconstruct the deep phylogenetic relationships among 46 species representing all major mantellid lineages, including 3760 basepairs (bp) of the mitochondrial gene fragments 12SrRNA (12S, 538 bp), 16SrRNA (16S, two fragments of 582 bp and 505bp), cytochrome b (cob, 988 bp), cytochrome oxidase subunit I (cox1, 625 bp), and of the nuclear rhodopsin exon 1 (289 bp) and RAG2 (816 bp) gene fragments; (ii) a 1172 bp mitochondrial data set of 16S, cob and cox1 sequences from all but three described mantellid species species (187 of the 190 described and valid species) plus 67 undescribed confirmed candidate species. The reduced-taxa data set was largely based on sequences used in other studies [31,56], complemented by additional sequences for crucial species (see Additional file 1, Table S1). In the all-taxa dataset, 16S sequences were mainly taken from previous work [24], whereas most sequences of cob and cox1 were newly determined.

PCRs were performed according to the reaction conditions and thermocycling protocols described previously [44,54,56,57]. Primers for the various genes were as specified in these previous publications (see Additional file 1, Table S2 for primer sequences). Sequencing reactions were performed with the forward primers and resolved on automated sequencers by Macrogen Inc., Korea for mitochondrial markers. Nuclear markers and ambiguous sequences of the mitochondrial markers were additionally sequenced with the reverse primers, with several sequencing reactions being repeated numerous times to obtain unambiguous results. The obtained electropherograms were manually edited and verified as mantellid DNA via BLAST searches. Alignments were generated with MEGA using the CLUSTALW algorithm [58] and refined manually. Gapped and hypervariable regions of the rRNA gene sequences were excluded from the analysis. Newly determined DNA sequences were submitted to Genbank (accession numbers JN132821-JN133276; for a complete list of GenBank accession numbers and a list of voucher specimens see Additional file 1, Table S1. For the reduced-taxa data set we used *Heterixalus variabilis *(a representative of the Hyperoliidae) as outgroup and included *Polypedates *spp. (Rhacophoridae, the sister group of the Mantellidae) as a hierarchical outgroup. For the all-taxa data set we defined *Polypedates *spp. as outgroup.

Best-fit models of evolution were constructed for the various character sets used in the partitioned analysis with MrModeltest [59] (Additional file 1, Table S3). To obtain the optimal partitioning strategy for the dataset, Bayesian tree searches were run for 20,000,000 generations for the reduced-taxa dataset and for 5,400,000 - 10,000,000 generations in the all-taxa dataset each with 2 runs and 4 chains (MrBayes V.3.1.2 [60]). Harmonic means were calculated using the sump command in MrBayes, with a conservative burn-in corresponding to the first 500,000 generations after assessing that stability of likelihood values had been reached in each case after much fewer generations. The partitioning strategies that explained the data set with the least random error were the maximum partition datasets in both analyses [61].

For the reduced-taxa data set, ML searches were conducted using the software RaxML V.7.0.0 (under the estimated best substitution model GammaInvar) [62], and the rapid bootstrapping algorithm was used with an estimated number of bootstraps [63]. Heuristic searches under MP were conducted for the reduced-taxa dataset in PAUP* [64], with 2000 bootstrap replicates. Characters in the MP searches were treated as unordered with equal weight. Gaps were treated as "missing"; multistate characters were interpreted as "uncertain". Trees were computed with random stepwise addition of taxa, and branch swapping was performed with the TBR (Tree-Bisection-Reconnection) algorithm, without limitation in the number of retained trees.

We then constrained the major lineages (subfamilies and relationships among some genera and subgenera) in the all-taxa dataset according to the optimal topology found for the reduced-taxa dataset, to enable a stable run of MrBayes under optimized computation time. A final Bayesian analysis was then performed for the all-taxa dataset running 30,000,000 generations for 58 days on a computing cluster at UC Berkeley and the 50%-majority consensus tree from this run after discarding the burnin was used as the preferred estimate of mantellid relationships.

For the all-taxa data set, we subsequently computed an ultrametric tree to estimate evolutionary node ages for ARC from the preferred Bayesian tree topology. We used the software Pathd8 to estimate a time-calibrated phylogeny [65] which computes ultrametric trees for large data sets. The rationale for using Pathd8 instead of more commonly used software like BEAST [66] or MultiDivtime [67] was two-fold: first, both alternative software crashed on our dataset during repeated trials. Second, the Pathd8 software has been introduced as especially suited for large datasets, being only less precise compared with penalized likelihood methods, but giving more sensible answers for extreme data sets. The reason for its faster performance with large data sets is that substitution rates are being smoothed locally, rather than simultaneously over the whole tree [65]. We used the estimated evolutionary split of the two undescribed species endemic to the Comoro island of Mayotte (*Blommersia *sp. 4, *Boophis *sp. 1) as fixed calibration points, which is estimated at 8.7 ma based on the age of the volcanic island of Mayotte [[44]; validated in [68]]. For adjustment of the deeper branches, we furthermore applied secondary age constraints, with divergence time estimates between mantellid genera based on the confidence intervals calculated in a previous study [69] (Additional file 1, Table S4). These secondary constraints were obtained from a estimation of divergence times on the basis of external calibration points and in general were fully congruent with other estimates of mantellid ages [e.g., [70]]. We also estimated divergence times without these secondary constraints and obtained a largely similar time frame for mantellid diversification, leaving us confident that these secondary constraints have not introduced any major bias in our analysis. In any case, our subsequent analyses of body and range size influences on diversification depend on relative, not absolute age of nodes, and thus are independent from possible inaccuracies that may remain in our estimates of absolute ages of diversification events.

We identified 53 pairs of mantellid sister species in the all-taxa phylogeny that were supported by high Bayesian posterior probabilities (>98). We did not consider sister species that had low Bayesian support values, or unknown ranges. Due to severe uncertainties in taxonomy and thus range estimations we also excluded well-supported sister species of the subgenera *Ochthomantis *(*Mantidactylus*) and *Pandanusicola *(*Guibemantis*). Because the two Comoroan species (*Boophis *sp. 1 and *Blommersia *sp. 4) likely originated by overseas dispersal (Vences et al., 2003), they were also excluded from subsequent calculations. The ages of all nodes separating pairs of sister species were extracted from the ultrametric tree obtained with Pathd8 and were tested for normal distribution with a one-tailed Kolmogorov-Smirnov test using STATISTICA (^© ^StatSoft, Tulsa, OK).

### Geographic data and analysis

We used point locality information for 242 species and candidate species in our phylogeny obtained from an extensive GIS-referenced database [29] to construct distribution maps with ArcView GIS (V.3.2a, Esri ^©^1992-2000). For species that were only known from one or two localities, we estimated species distribution areas by assigning buffer zones around these localities. The estimation of small range sizes is crucial for our paper, so we conducted all analyses using two different estimates for these buffer zones: a rather large one of 25 km radius versus a small quasi-zero one of 17 m radius. Comparing results obtained with both buffer zone estimates ensures the robustness of our results without knowing the exact extent of range size of these species, although according to our own observations [29], the larger estimate is probably an overestimation of the range sizes of microendemic species. However, we emphasize that one or two-locality species do not equal limited sampling effort: In most cases, these are well-identifiable species that have not been found elsewhere despite important survey efforts in Madagascar over the past 20 years. Subclades with taxonomic or range uncertainties (e.g., in the subgenera *Ochthomantis *and *Pandanusicola*) were excluded from analysis). For species that are known from more than two localities, minimum convex polygons (MCPs) were taken as estimate for real species distribution area size. MCPs span the whole area between two occurrence records of a species, disregarding climatic and habitat differences and possible range discontinuities. However, for the understanding of past evolutionary processes, we consider these analyses as adequate because the current distribution of a species alone may be misleading. For instance, if a species has currently a very fragmented range, in the past it must have dispersed from one of its current range fragments to the other and thus occupied a much larger and more continuous range in which it may have co-occurred broadly with other species. Especially in the instance of limited extent of range size and locality records MCPs may therefore be more realistic than fine-scale mapping or modeling on the basis of habitat data as has been applied for other purposes [71]. Furthermore, distribution area modeling is not advised for species with less than five locality records. We call the dataset containing MCPs and large buffer zones "Range Size A dataset" (RSA) and the dataset containing MCPs and small buffer zones "Range Size B dataset" (RSB). For each MCP, we furthermore determined the centroid using the xtools^® ^extension in ArcView. Centroids for two-locality species were estimated half way between these localities. Range proximity among all mantellid species (measured as distance between polygon centroids in km) was determined by calculating a Euclidean distance matrix of polygon centroids using the ArcView "distance matrix" extension (^© ^Jenness, J., 2005), and age-range correlation [37] was calculated based on range overlap in km^2^, transformed to overlap in percent of the smaller of each two polygons. We automatized the computation of range overlap in km^2 ^and the resulting percentage of range overlap with a script in ArcView (^© ^Schmalstieg, K.J., 2007). Centroid distances can be measured in species pairs characterized by fully allopatric distributions as well as in those with partly overlapping ranges. We furthermore preferred using centroid distances because these are less heavily affected by possible sampling gaps than are distances between range borders.

Under the hypothesis of peripatric speciation, range size differences are thought to be initially large, a pattern that can but does not necessarily have to dilute over time. Under initially large range size contrasts, we expect either a negative or no correlation between range size differences and evolutionary age of sister species. We tested for this expected, possibly triangular pattern of peripatric speciation in mantellids using quantile regression in R [72,73] on sister species pairs. Quantile regression accounts for the fact that more than a single slope can describe the relationship between a response variable and a predictor and can discover predictive relationships between variables in cases where there is no or only a weak relationship between the variable means. It allows computing regressions of different sets of the data (e.g., the 90% quantile is the regression slope above 90% of the data points). Multiple slopes are used to describe the relationship between variables that would be missed by other regression models [73].

Due to limited number of localities and small extent of distribution area for many species, environmental niche modeling did not make sense for our dataset. Instead, 21 climatic variables for each locality per species were extracted from the WORLDCLIM climatic maps (1 km × 1 km resolution, interpolated from lower resolution) [71,74]. To correct for co-variation among these 21 climatic variables, a Principal Component Analysis (PCA) was performed in Varimax-rotated coordinate system, yielding four factors (PCs) with Eigenvalues >1 (Additional file 1, Tables S5, S6). The highest Eigenvalues were 10.4 for PC1 and 5.9 for PC2, followed by lower Eigenvalues for PCs 3 and 4 (2.4, and 1.1, respectively). From these four factors we calculated squared Mahalanobis distances (which we chose because of multiple locality information per species) between all mantellid species and extracted the data for mantellid sister species with significant branch support from this triangular matrix. These bioclimatic distances served as a covariate of spatial distance.

### Testing MEP hypotheses: body size, range size, and diversification

Maximal Snout-Vent Length (SVL) of males has been used as a proxy for body size of frog species before [75,76]. We compiled values for maximal male SVL for 249 mantellid species and candidate species from Glaw and Vences (2007) and complemented these with own, unpublished data. For the complete list of SVL data, see Additional file 1, Table S1. We computed range size frequency distributions for all mantellids, for mantellid sister species using STATISTICA (Tulsa, OK), and counted the number of species with non-overlapping ranges (allopatric species pairs) and partially or fully overlapping ranges (sympatric species pairs).

A first set of statistical tests was carried out based on the 53 well-supported pairs of sister species as identified in our phylogenetic analysis. We used these pairs as independent data points in tip-based phylogenetic contrast method (PCM) approaches. We computed standardized tip contrasts for range size and body size between them ((RSA1 - RSA2)/SQRT(branch length 1 + branch length 2); ((RSB1 - RSB2)/SQRT(branch length 1 + branch length 2); (SVL1 - SVL2)/SQRT(branch length 1 + branch length 2)) [22]. The branch lengths were taken from the Bayesian phylogeny before ultrametric correction.

We tested whether contrasts in SVL are also a predictor for contrasts of range size in mantellid sister species pairs using univariate regression analyses through the origin. We furthermore correlated range size contrasts with body size contrasts to infer whether small values in both are associated with each other.

To infer whether small range size and/or body size contrasts are pronounced in proximate and recently diverged lineages we performed a factorial regression analysis through the origin. This analysis estimated the effect of evolutionary age, range proximity (expressed as centroid distance), bioclimatic distance (as a covariate to spatial distance, for spatial structure of the climatic niche) and their respective interaction terms on SVL and range size (RSA and RSB) contrasts between sister species pairs. Older species pairs with larger range sizes and/or larger body size were expected to more likely have larger range size contrasts and larger body size contrasts, accounting for the possibility of (potentially asymmetric) post-speciation range shifts or range size changes.

Additional to the results obtained by the sister species tip contrasts we applied phylogenetic comparative methods (PCM) that utilize the whole tree. Effects of SVL on RSA/RSB as well as the effect of these characters on the mitochondrial substitution rate of the all-taxa phylogeny were determined using the software CoEvol [33]. The approximated synonymous substitution rate (dS) and the continuous characters were jointly modeled as a multivariate Brownian diffusion process of unknown covariance matrix [33] on the concatenated dataset with fixed divergence times. The covariance matrix, phylogenetic variation of the substitution rates, and the continuous characters are then jointly estimated by a Bayesian MCMC process. Because all parameters are modeled in a single multivariate distribution process, substitution rates and morphological traits can be analyzed in a single statistical framework [33].

We tested the effect of body size and range size on clade diversity using a second PCM implemented in the software MacroCAIC [32], which is a modified version of comparative analysis by independent contrasts [22,77]. Species richness contrasts (RRD) as implemented in MacroCAIC are positive, when clades containing species with large values of the inherited character in question are more species-rich than their sister clade, leading to a positive correlation between variable contrasts and richness contrasts [32]. In our example we expect negative richness contrasts in clades with high SVL, leading to a negative correlation between SVL contrast and richness contrast (defining the MEP). To determine the effect of SVL changes on species richness, we performed a regression through the origin for the contrasts produced by MacroCAIC using STATISTICA [11].

## Authors' contributions

KCW, MV and DRV conceived the study. KCW performed all lab work and analyses. FG and DRV provided crucial samples and contributed to data interpretation. KCW and MV wrote the paper. All authors read and approved the final manuscript.

## Supplementary Material

Additional file 1 Includes a full list of voucher specimens, Genbank accession numbers, primer sequences, a phylogenetic tree obtained on the basis of the reduced-taxa data set, as well as additional tables with more detailed results of several statistical analyses. Click here for file

## References

[B1] Losos JB Glor R Phylogenetic comparative methods and the geography of speciation Trends Ecol Evol 2003 18 220 227 10.1016/S0169-5347(03)00037-5

[B2] Yoder JB Clancey E Des Roches S Eastman JM Gentry L Godsoe W Hagey TJ Jochimsen D Oswald BP Robertson J Sarver BAJ Schenk JJ Spear SF Harmon LJ Ecological opportunity and the origin of adaptive radiations J Evol Biol 2010 23 1581 1596 10.1111/j.1420-9101.2010.02029.x 20561138

[B3] Losos JB Adaptive radiation, ecological opportunity, and evolutionary determinism Am Nat 2010 175 623 639 10.1086/652433 20412015

[B4] Via S Sympatric speciation in animals: the ugly duckling grows up Trends Ecol Evol 2001 16 381 390 10.1016/S0169-5347(01)02188-7 11403871

[B5] Butlin RK Galindo J Grahame JW Sympatric, parapatric or allopatric? The most important way to classify speciation Phil Trans Roy Soc B 2008 363 2997 3007 10.1098/rstb.2008.0076 PMC2607313 18522915

[B6] Simpson GG Tempo and Mode in Evolution 1949 Columbia University Press, New York

[B7] Simpson GG The Major Features of Evolution 1953 Columbia University Press, New York

[B8] Sanderson MJ Donoghue MJ Reconstructing shifts in diversification rates on phylogenetic trees Trends Ecol Evol 1996 11 15 20 10.1016/0169-5347(96)81059-7 21237745

[B9] Peters RH The Ecological Implications of Body Size Cambridge Studies in Ecology 1983 Cambridge University Press, Cambridge

[B10] Gaston KJ Blackburn TM Range size-body size relationships: evidence of scale dependence Oikos 1996 75 479 485 10.2307/3545889

[B11] Garland TJ Harvey PH Ives AR Procedures for the analysis of comparative data using phylogenetically independent contrasts Syst Biol 1992 41 18 32

[B12] Giannini NP Goboloff PA Delayed-response phylogenetic correlation: an optimization-based method to test covariation of continuous characters Evolution 2010 64 1885 1898 20100217 10.1111/j.1558-5646.2010.00956.x

[B13] Streelman JT Danley PD The stages of evolutionary radiation Trends Ecol Evol 2003 18 126 131 10.1016/S0169-5347(02)00036-8

[B14] Williams EE The origin of faunas: evolution of lizard congeners in a complex island fauna: a trial analysis Evol Biol 1972 6 47 89

[B15] Stanley SM An explanation for Cope's rule Evolution 1973 27 1 26 10.2307/2407115 28563664

[B16] Gaston KJ Blackburn TM Pattern and process in macroecology 2000 Oxford: Blackwell Science

[B17] David C Orme L Isaac NJB Purvis A Are most species small? Not within species-level phylogenies Proc Roy Soc Lond B 2002 269 1279 1287 10.1098/rspb.2002.2003 PMC1691029 12065045

[B18] Meiri S Evolution and ecology of lizard body sizes Global Ecol Biogeogr 2008 17 724 734 10.1111/j.1466-8238.2008.00414.x

[B19] Lindsey CC Body size of poikilotherm vertebrates at different latitudes Evolution 1966 20 456 465 10.2307/2406584 28562908

[B20] Gardezi T da Silva J Diversity in relation to body size in mammals: a comparative study Am Nat 1999 153 110 123 10.1086/303150 29578766

[B21] Van Bocxlaer I Loader SP Roelants K Biju SD Menegon M Bossuyt F Gradual adaptation toward a range-expansion phenotype initiated the global radiation Science 2010 327 679 682 10.1126/science.1181707 20133569

[B22] Felsenstein J Phylogenies and the comparative method Am Nat 1985 125 1 15 10.1086/284325

[B23] Fitzpatrick BM Turrelli M The geography of mammalian speciation: mixed signals from phylogenies and range maps Evolution 2006 60 601 615 16637504

[B24] Vieites DR Wollenberg KC Andreone F Köhler J Glaw F Vences M Vast underestimation of Madagascar's biodiversity evidenced by an integrative amphibian inventory Proc Natl Acad Sci USA 2009 106 8267 8272 10.1073/pnas.0810821106 19416818 PMC2688882

[B25] Vences M Köhler J Global diversity of amphibians (Amphibia) in freshwater Hydrobiologia 2008 595 569 580 10.1007/s10750-007-9032-2

[B26] Santos JC Coloma LA Summers K Caldwell JP Ree R Cannatella DC Amazonian amphibian diversity is primarily derived from Late Miocene Andean lineages PloS Biology 2009 7 e1000056 10.1371/journal.pbio.1000056 PMC2653552 19278298

[B27] Heinicke MP Duellmann WE Hedges SB Major Caribbean and Central American frog faunas originated by ancient oceanic dispersal Proc Natl Acad Sci USA 2007 104 10092 10097 10.1073/pnas.0611051104 17548823 PMC1891260

[B28] Van der Meijden A Vences M Hoegg S Meyer A A previously unrecognized radiation of ranid frogs in southern Africa revealed by nuclear and mitochondrial DNA sequences Mol Phyl Evol 2005 37 674 685 10.1016/j.ympev.2005.05.001 15975829

[B29] Glaw F Vences M A Field Guide to the Amphibians and Reptiles of Madagascar 2007 3 Vences and Glaw, Cologne

[B30] Stuart S Hoffmann M Chanson J Cox N Berridge R Ramani P Young B (eds) Threatened Amphibians of the world 2008 Lynx Edicions, IUCN, and Conservation International, Barcelona, Spain: Gland, Switzerland, and Arlington, Virginia, USA

[B31] Glaw F Vences M Phylogeny and genus-level classification of mantellid frogs Org Divers Evol 2006 6 236 253 10.1016/j.ode.2005.12.001

[B32] Lartillot N Poujol R A phylogenetic model for investigating correlated evolution of substitution rates and continuous phenotypic characters Mol Biol Evol 2011 28 729 744 10.1093/molbev/msq244 20926596

[B33] Agapow PM Isaac NJB MacroCAIC: revealing correlates of species richness by comparative analysis Divers Distrib 2002 8 41 43 10.1046/j.1366-9516.2001.00121.x

[B34] Wilmé L Goodman SM Ganzhorn JU Biogeographic evolution of Madagascar's microendemic biota Science 2006 312 1063 1065 10.1126/science.1122806 16709785

[B35] Rull V Speciation timing and Neotropical biodiversity: the Tertiary-Quaternary debate in the light of molecular phylogenetic evidence Mol Ecol 2008 17 2722 2729 10.1111/j.1365-294X.2008.03789.x 18494610

[B36] Townsend TM Vieites DR Glaw F Vences M Testing species-level diversification hypotheses in Madagascar: the case of microendemic *Brookesia *leaf chameleons Syst Biol 2009 58 461 656 10.1093/sysbio/syp042 20525615

[B37] Lynch JD D. Otte, J. A. Endler The gauge of speciation: on the frequencies of modes of speciation Speciation and its consequences 1989 Sinauer, Sunderland 527 553

[B38] Berlocher SH J. D. Howard, S. H. Berlocher Can sympatric speciation be proven from biogeographic and phylogenetic evidence? Endless Forms: Species and Speciation 1998 Oxford University Press, New York 99 113

[B39] Coyne JA Price TD Little evidence for sympatric speciation in island birds Evolution 2000 54 2166 2171 11209793 10.1111/j.0014-3820.2000.tb01260.x

[B40] Bolnick DI Fitzpatrick BM Sympatric speciation: models and empirical evidence Annu Rev Ecol Syst 2007 38 459 487 10.1146/annurev.ecolsys.38.091206.095804

[B41] Maurer BA Brown JH Rusler RD The micro and macro in body size evolution Evolution 1992 46 939 953 10.2307/2409748 28564414

[B42] Chesser RT Zink RM Modes of speciation in birds: a test of Lynch's method Evolution 1994 48 490 497 10.2307/2410107 28568302

[B43] Barraclough TG Vogler AP Detecting the geographical patterns of speciation from species-level phylogenies Am Nat 2000 155 419 434 10.1086/303332 10753072

[B44] Vences M Vieites DR Glaw F Brinkmann H Kosuch J Veith M Meyer A Multiple overseas dispersal in amphibians Proc Roy Soc Lond B 2003 270 2435 2442 10.1098/rspb.2003.2516 PMC1691525 14667332

[B45] Glaw F Vences M A new cryptic species of the *Mantidactylus boulengeri *group with a divergent vocal sac structure Amphibia-Reptilia 2002 23 293 304 10.1163/15685380260449171

[B46] Wollenberg KC Harvey J First assessment of the territorial vocal behaviour in a Malagasy leaf litter frog (*Gephyromantis thelenae*) Herpetology Notes 2010 3 141 150

[B47] Mayr E A. R. I. Liss Processes of speciation in animals Mechanisms of Speciation 1982 Alan R. Liss Inc., New York 1 19

[B48] Barraclough TG Nee S Phylogenetics and speciation Trends Ecol Evol 2001 16 391 399 10.1016/S0169-5347(01)02161-9 11403872

[B49] Eo SH DeWoody A Evolutionary rates of mitochondrial genomes correspond to diversification rates and to contemporary species richness in birds and reptiles Proc Roy Soc Lond B 2010 277 3587 3592 10.1098/rspb.2010.0965 PMC2982251 20610427

[B50] Phillimore AB Price TD R. K. Butlin, J. R. Bridle, D. Schluter Ecological influences on the temporal pattern of speciation Speciation and patterns of diversity 2009 Cambridge University Press, Cambridge 240 256

[B51] White EP Relationships between body size and abundance in ecology Trends Ecol Evol 2007 22 323 330 10.1016/j.tree.2007.03.007 17399851

[B52] Schluter D The Ecology of Adaptive Radiation 2000 Oxford Univ. Press, Oxford

[B53] Hutchinson GE MacArthur RH A theoretical ecological model of size distributions among species of animals Am Nat 1959 138 1478 1512

[B54] Vences M Thomas M Bonnett RM Vieites DR Deciphering amphibian diversity through DNA barcoding: chances and challenges Phil Trans Roy Soc Lond B 2005 360 1859 1868 10.1098/rstb.2005.1717 PMC1609216 16221604

[B55] Padial JM Miralles A de la Riva I Vences M The integrative future of taxonomy Front Zool 2010 7 article 16 10.1186/1742-9994-7-16 PMC2890416 20500846

[B56] Vences M Wahl-Boos G Hoegg S Glaw F Spinelli Oliveira E Meyer A Perry S Molecular systematics of mantelline frogs from Madagascar and the evolution of their femoral glands Biol J Linn Soc 2007 92 529 539 10.1111/j.1095-8312.2007.00859.x

[B57] Hoegg S Vences M Brinkmann H Meyer A Phylogeny and comparative substitution rates of frogs inferred from three nuclear genes Mol Biol Evol 2004 21 1188 1200 10.1093/molbev/msh081 14963093

[B58] Kumar S Dudley J Nei M Tamura K MEGA: A biologist-centric software for evolutionary analysis of DNA and protein sequences Brief Bioinf 2008 9 299 306 10.1093/bib/bbn017 PMC2562624 18417537

[B59] Nylander JAA MrModeltest v2. Program distributed by the author 2004 Evolutionary Biology Center, Uppsala University

[B60] Ronquist F Huelsenbeck JP MrBayes3: Bayesian phylogenetic inference under mixed models Bioinf 2003 19 1572 1574 10.1093/bioinformatics/btg180 12912839

[B61] Brandley MC Schmitz A Reeder TW Partitioned Bayesian analyses, partition choice, and the phylogenetic relationships of scincid lizards Syst Biol 2005 54 373 390 10.1080/10635150590946808 16012105

[B62] Stamatakis A RAxML-VI-HPC: Maximum Likelihood-based phylogenetic analyses with thousands of taxa and mixed models Bioinf 2006 22 2688 2690 10.1093/bioinformatics/btl446 16928733

[B63] Stamatakis A Hoover P Rougemont J A rapid bootstrap algorithm for the RAxML web-servers Syst Biol 2008 75 758 771 10.1080/10635150802429642 18853362

[B64] Swofford DL PAUP* Phylogenetic Analysis Using Parsimony (*and Other Methods). Version 4 2002 Sunderland, MA: Sinauer Associates

[B65] Britton T Anderson CL Jacquet D Lundqvist S Bremer K Estimating divergence times in large phylogenetic trees Syst Biol 2007 56 741 752 10.1080/10635150701613783 17886144

[B66] Drummond AJ Rambaut A BEAST: Bayesian evolutionary analysis by sampling trees BMC Evol Biol 2007 7 214 10.1186/1471-2148-7-214 17996036 PMC2247476

[B67] Thorne JL Kishino H Divergence time and evolutionary rate estimation with multilocus data Syst Biol 2002 51 689 702 10.1080/10635150290102456 12396584

[B68] San Mauro D Vences M Alcobendas M Zardoya R Meyer A Initial diversification of living amphibians predated the breakup of Pangaea Am Nat 2005 165 590 599 10.1086/429523 15795855

[B69] Kurabayashi A Sumida M Yonekawa H Glaw F Vences M Hasegawa M Phylogeny, recombination, and mechanisms of stepwise mitochondrial genome reorganization in mantellid frogs from Madagascar Mol Biol Evol 2008 25 874 891 10.1093/molbev/msn031 18263605

[B70] Roelants K Gower DJ Wilkinson M Loader SP Biju SD Guillaume K Moriau L Bossuyt F Global patterns of diversification in the history of modern amphibians Proc Natl Acad Sci USA 2007 104 887 892 10.1073/pnas.0608378104 17213318 PMC1783409

[B71] Kremen C Cameron A Moilanen A Phillips SJ Thomas CD Beentje H Dransfield J Fisher BL Glaw F Good TC Harper GJ Hijmans RJ Lees DC Louis E Jr Nussbaum RA Raxworthy CJ Razafimpahanana A Schatz GE Vences M Vieites DR Zjhra ML Aligning conservation priorities across taxa in Madagascar with high-resolution planning tools Science 2008 320 222 226 10.1126/science.1155193 18403708

[B72] Koenker R Basset G Regression quantiles Econometrica 1978 46 33 50 10.2307/1913643

[B73] Cade BS Noon BR A gentle introduction to quantile regression for ecologists Front Ecol Evol 2003 1 412 420

[B74] Hijmans RJ Cameron SE Parra JL Jones PG Jarvis A Very high resolution interpolated global terrestrial climate surfaces Int J Climatol 2005 25 1965 1978 10.1002/joc.1276

[B75] Moen DS Smith SA Wiens JJ Community assembly through evolutionary diversification and dispersal in Middle American treefrogs Evolution 2009 63 3228 3247 10.1111/j.1558-5646.2009.00810.x 19663988

[B76] Moen DS Wiens JJ Phylogenetic evidence for competitively driven divergence: body-size evolution in Caribbean treefrogs (Hylidae: *Osteopilus*) Evolution 2009 63 195 214 10.1111/j.1558-5646.2008.00538.x 19055679

[B77] Purvis A Rambaut A Comparative analysis by independent contrasts (CAIC): an Apple Macintosh application for analysing comparative data Comp Appl Biosci 1995 11 247 251 7583692 10.1093/bioinformatics/11.3.247

[B78] Maddison WP Maddison DR Mesquite: a modular system for evolutionary analysis 2006 http://mesquiteproject.org/mesquite/mesquite.html Version 2.74

