# Supplementary material for: Speciation in little: the role of range and body size in the diversification of Malagasy mantellid frogs
Source: BMC Evol Biol. 2011 Jul 21;11:217. doi: 10.1186/1471-2148-11-217 (PMC3199771; doi:10.1186/1471-2148-11-217)

**Online Supplementary Material**

To the manuscript:

**Speciation in little: the role of range and body size in the diversification of Malagasy mantellid frogs**

Katharina C. Wollenberg*^1^, David R. Vieites^2^, Frank Glaw^3^ and Miguel Vences^1^

7 Supplementary tables

3 Supplementary figures

Supplementary Table S1. Locality information, voucher specimens and GenBank accession numbers used in molecular phylogeny. Species names used in the phylogeny correspond to those in Vieites et al. (2009), working names of species currently under description are given in quotation marks. Number of localities can be obtained from Vieites et al. (2009). Note that in most cases, the sequences listed refer to the same voucher specimen which preferably is part of the type series of the respective species or was collected near the type locality. In a few cases, sequences of different conspecific specimens from the various genes were combined for analysis; voucher specimen details are always found in the respective GenBank record.

| **Name as in tree** | **Locality of sequenced specimen** | **Voucher number** | **Genbank accession numbers: 16S, cob, cox1, if sequenced 12S, 16S2, Rhod, RAG2** | **Max. male SVL (in mm)** | **Range size in km^2^**  **(or RSA/RSB estimate)** |
| --- | --- | --- | --- | --- | --- |
| *Aglyptodactylus laticeps* | Kirindy | ZSM 199/2003 | AY847959, JN132844, JN133053 | 45 | RSA/RSB |
| *Aglyptodactylus madagascariensis* | Montagne d'Ambre | ZSM 203/2004 | AY847979, JN132845, JN133054, AY341678, AF249007, AF249103, DQ019516 | 41 | 78798.64 |
| *Aglyptodactylus securifer* | Bemaraha | ZSM 14/2006 | FJ559108, JN132846, JN133055 | 35 | 42852.35 |
| *Aglyptodactylus* sp. 2 | Maroantsetra | MVTIS 2002A70 | FJ559109, JN132847, JN133056 | 35 | 1291.781 |
| *Aglyptodactylus* sp. 3 | Ranomafana | ZMA 20259 | AY8487999, JN132848, JN133057 | 35 | 1465.675 |
| *Blommersia blommersae* | Andasibe | UADBA (FGMV 2000.65) | DQ235418, GU983151, GU983137, AY341584, AY341638, AY341770, JN132824 | 21 | 102249.4 |
| *Blommersia* *dejongi* | Toamasina | ZSM 455/2006 (ZCMV 3233) | FJ559117, HM776663, HM776666 | 20.8 | 1602.305 |
| *Blommersia domerguei* | Ranomafana (Ambatovory) | ZMA 20056 | AY848071, GU983150, GU983136 | 17 | 30381.7 |
| *Blommersia* *galani* | Nosy Boraha | ZSM 453/2006 (ZCMV 3232) | FJ559116, HM776662, HM776665 | 24.2 | 24.84194 |
| *Blommersia grandisonae* | Ambato | ZFMK 66669 | FJ559115, GU983147, GU983133 | 23 | 102050.7 |
| *Blommersia kely* | Antoetra | ZMA 19551 | AY848078, GU983149, GU983135 | 16 | 1055.783 |
| *Blommersia sarotra* | Mandraka | ZSM 354/2000 | AF317687, GU983148, GU983134, AY341588, AY341643, AY341773, JN132825 | 15.9 | 1266.939 |
| *Blommersia* sp. 2 "variabilis" | Maroantsetra | MVTIS 2002A10 | AY848104, JN132850, JN133059 | 24.7 | 2061.881 |
| *Blommersia* sp. 4 | Mayotte (Comoro Islands) | ZSM 652/2000 | AY341639, JN132851, JN133060, AY341585, AY341639, AY323742, AY323794 | not analyzed | not analyzed |
| *Blommersia* sp. 5 | Ranohira | ZSM 22/2004 | AY848031, JN132852, JN133061 | 26 | 21177.76 |
| *Blommersia* sp. 11 | Manombo | ZCMV 5432 | FJ559120, JN133052, JN133276 | 20.1 | no data |
| *Blommersia wittei* | Montagne d'Ambre | ZSM 879/2003 | AY848105, GU983146, GU983132, AY341641, AY341586, AY323743, AY323795 | 26 | 75954.24 |
| *Boehmantis microtympanum* | Andohahela | ZSM 79/2004 | AY848203, JN132853, JN133062, DQ235455, DQ235451, DQ235445, JN132826 | 80 | 2633.246 |
| *Boophis albilabris* | Manongarivo | UADBA (FGMV 2002.807) | AY848556, JN132855, JN133064 | 73 | 153150.6 |
| *Boophis albipunctatus* | Manantantely | ZSM 157/2004 | AY848446, JN132857, JN133066 | 33 | 12917.81 |
| *Boophis andohahela* | Andohahela | ZSM 157/2005 (FGZC 2372) | FJ559121, JN132858, JN133067 | 30 | 4123.763 |
| *Boophis* *andrangoloaka* | Ambohitantely | ZSM 5190/2005 (FGZC 2139) | FJ559150, JN132910, JN133118 | 29 | RSA/RSB |
| *Boophis andreonei* | Manongarivo | ZMA (FGMV 2002.806) | AY848449, JN132859, JN133068 | 33 | 645.8905 |
| *Boophis anjanaharibeensis* | Marojejy | ZSM 100/2005 (FGZC 2762) | FJ559122, JN132860, JN133069 | 36 | 3925.027 |
| *Boophis ankaratra* | Manjakatompo | ZSM 367/2000 | AJ315911, JN132861, JN133070 | 28 | 33996.2 |
| *Boophis* *arcanus* | Mahakajy | UADBA 24299 | AY848632, JN132896, JN133105 | 32 | RSA/RSB |
| *Boophis axelmeyeri* | Manongarivo | UADBA (FGMV 2002.809) | DQ118668, JN132864, JN133073 | 43 | 447.155 |
| *Boophis baetkei* | Forêt d'Ambre | ZSM 2051/2007 (FGZC 1391) | EU314954, ---, --- | no data | RSA/RSB |
| *Boophis blommersae* | Montagne d'Ambre | ZSM 906/2003 | AY883982, JN132865, JN133074 | 27 | 298.1033 |
| *Boophis boehmei* | Andasibe | UADBA (FGMV 2001.1205) | AY848559, DQ235433, JN133075, AY341669, AY341612, AY341798, EF100488 | 29 | 3974.711 |
| *Boophis bottae* | Andasibe | ZSM 344/2000 | AJ314817, JN132867, JN133077 | 24 | 4483.971 |
| *Boophis brachychir* | Manongarivo | UADBA (FGMV 2002.702) | AY848538, ---, JN133078 | 50 | 1751.357 |
| *Boophis burgeri* | Andasibe | UADBA (FGMV 2001.1246) | AY848566, JN132869, JN133080 | 38 | 2260.617 |
| *Boophis* *calcaratus* | Ambavaniasy | NMBE 862/95 | FJ559134, JN132918, JN133126 | 29.5 | 2260.617 |
| *Boophis doulioti* | Bemaraha | ZSM 29/2006 | FJ559123, JN132870, JN133081, AY341664, AF249009, AF249105, DQ019519 | 42 | 278329.1 |
| *Boophis elenae* | Ranomafana (Maharira) | UADBA 24141 | AY848470, JN132871, JN133082 | 40 | 4297.656 |
| *Boophis englaenderi* | Marojejy | ZSM 418/2005 (ZCMV 856) | FJ559124, JN132873, JN133084 | 35 | 757.6793 |
| *Boophis* *entingae* | Montagne d'Ambre | ZSM 899/2003 | AY848563, JN132868, JN133079 | 50 | 3577.24 |
| *Boophis erythrodactylus* | Mandraka | ZSM 344/2000 | AJ314814, JN132874, JN133085 | 25 | 3925.027 |
| *Boophis feonnyala* | Andasibe | ZSM 313/2000 | AJ315922, JN132875, JN133086 | 25 | RSA/RSB |
| *Boophis goudotii* | Antoetra | ZMA 19544 | AY848570, JN132876, DQ116466 | 70 | 46243.28 |
| *Boophis guibei* | Andasibe | ZSM 348/2000 | DQ116467, JN132877, DQ116467 | 40 | 10210.04 |
| *Boophis haematopus* | Andohahela | ZSM 120/2004 | AY848633, JN132878, JN133087 | 28 | 186.3146 |
| *Boophis* *haingana* | Andohahela | ZSM 5109/2005 (FGZC 2390) | FJ559142, JN132862, JN133071 | 28 | 3192.19 |
| *Boophis idae* | Andasibe | ZSM 45/2002 | AY848481, JN132879, JN133088, AY341666, AY341609, AY341795, EF100489 | 36 | 15650.42 |
| *Boophis jaegeri* | Nosy Be | ZSM 587/2001 | FJ559125, JN132880, JN133089 | 31 | 173.8936 |
| *Boophis laurenti* | Andringitra | ZSM 727/2001 | AY848575, JN132881, JN133090 | 33 | 12.42097 |
| *Boophis liami* | Vohidrazana | ZSM 310/2000 | AJ315921, JN132882, JN133091 | 21 | 202.4618 |
| *Boophis lichenoides* | Sahavontsira | FAZC 5679 | FJ664184, --, JN133092 | 43 | 28754.55 |
| *Boophis lilianae* | Ifanadiana | ZSM 201/2006 (ZCMV 2864) | EU314953, JN132907, JN133115 | 18.3 | RSA/RSB |
| *Boophis* *luciae* | Ranomafana (Ambatolahy) | ZMA 20306 | AY848443, JN132917, JN133125 | 31.3 | 17116.1 |
| *Boophis luteus* | Andasibe | UADBA (FGMV 2000.063) | AJ315916, JN132884, JN133094, AY341671, AY341614, AY341800, EF100490 | 40 | 79245.8 |
| *Boophis madagascariensis* | Ranomafana | ZMA (ZCMV 344) | AY848585, JN132885, JN133095 | 65 | 143598.9 |
| *Boophis majori* | Ranomafana (Maharira) | ZMA 20068 | AY848586, JN132887, JN133097 | 24 | 1664.41 |
| *Boophis mandraka* | Mandraka | ZSM 346/2000 | AJ315921, --- | 26 | RSA/RSB |
| *Boophis mandraka* | Ambatolahy | ZCMV 5332 | FJ559126, JN132889, JN133099 | 23 | RSA/RSB |
| *Boophis marojezensis* | Marojejy | ZSM 108/2005 (FGZC 2857) | FJ559127, JN132892, JN133102 | 27 | 78798.64 |
| *Boophis* *miadana* | Andohahela | ZSM 5108/2005 (FGZC 2389) | FJ559141, JN132863, JN133072 | 28 | RSA/RSB |
| *Boophis microtympanum* | Ambohitantely | ZSM 112/2005 (FGZC 2200) | FJ559129, JN132893, JN133103, AY341670, AY341613, AY341799, EF100491 | 30 | 34629.67 |
| *Boophis miniatus* | Manantantely | ZSM 142/2004 | AY848639, JN132895, -- | 32 | 111.7887 |
| *Boophis* *obscurus* | Ranomafana (Ranomafanakely) | ZMA 20219 | AY848606, JN132902, DQ116471 | 81.9 | 30009.07 |
| *Boophis occidentalis* | Near Antoetra - unconfirmed locality | ZSM 44/2002 | AY341677, JN132897, JN133106, AY341677, AY341620, AY341806, EF100492 | 52.4 | 29015.39 |
| *Boophis opisthodon* | Cap Est | ZFMK 70480 | AF215331, JN132899, JN133108 | 57 | 43796.35 |
| *Boophis pauliani* | Andasibe | ZSM 345/2000 | AJ315924, JN132900, JN133109, EF100469, EF100476, EF100482, EF100493 | 23 | 18631.46 |
| *Boophis* sp. aff. *pauliani* | Tolagnaro | ZSM 159/2002 | AY848493, ---, --- | 23 | RSA/RSB |
| *Boophis periegetes* | Andohahela | ZSM 114/2005 (FGZC 2430) | FJ559130, JN132901, JN133110 | 75 | 1478.096 |
| *Boophis picturatus* | An'Ala | ZSM 272/2006 (ZCMV 1457) | FJ559131, JN132903, JN133111 | 33 | 8160.578 |
| *Boophis* *piperatus* | Ranomafana | ZSM 377/2004 (ZCMV 320) | AY848627, JN132911, JN133119 | 29.6 | 1962.514 |
| *Boophis* *praedictus* | Vevembe | ZMA 20131 | AY848528, JN132856, JN133065 | 73 | 3925.027 |
| *Boophis pyrrhus* | Ifanadiana | UADBA 24315 | AY848645, JN132904, JN133112 | 32 | 49895.04 |
| *Boophis* *quasiboehmei* | Ranomafana | ZSM 227/2006 (ZCMV 3045) | FJ559139, JN132866, JN133076 | 35 | 2372.406 |
| *Boophis rappiodes* | Andasibe | UADBA 2000.59 | AJ314816, JN132905, JN133113 | 25 | 17053.99 |
| *Boophis reticulatus* | Maharira | ZMA (ZCMV 211) | AY848613, JN132908, JN133116 | 35 | 38666.48 |
| *Boophis rhodoscelis* | Ranomafana | ZMA (ZCMV 316) | AY848620, JN132909, JN133117 | 28.1 | 13986.01 |
| *Boophis* *roseipalmatus* | Montagne d’Ambre | ZSM 898/2003 | AY848577, JN132886, JN133096 | 65 | 3838.08 |
| *Boophis rufioculis* | An'Ala | 2002.GA272 | AY848623, JN132912, JN133120 | 35 | 2074.302 |
| *Boophis sambirano* | Manongarivo | ZSM 810/2003 | AY848544, JN132914, JN133122 | 24 | 3664.187 |
| *Boophis sandrae* | Ranomafana | ZMA 20133 | AY848442, JN132872, JN133083 | 48.2 | 8831.311 |
| *Boophis schuboeae* | Ranomafana | ZFMK 62907 | AJ315912, JN132915, JN133123 | 26 | 2111.565 |
| *Boophis septentrionalis* | Montagne d'Ambre | ZSM 900/2003 | AY848505, JN132916, JN133124 | 37 | 15712.53 |
| *Boophis sibilans* | Andasibe | ZSM 39/2002 | DQ235435, DQ235435, DQ116475 | 30 | 32654.73 |
| *Boophis solomaso* | Ambavaniasy | NMBE 1046008 | AJ315920, JN132919, JN133127 | 22 | 1962.514 |
| *Boophis* sp. 1 | Mayotte (Comoro Islands) | ZSM 658/2000 | FJ559133, JN132854, JN133063 | not analyzed | not analyzed |
| *Boophis* sp. 8 | Ranomafana (Maharira) | ZMA 20193 | AY848535, JN132913, JN133121 | 31 | RSA/RSB |
| *Boophis* sp. 27 | Masoala | FAZC 7805 | FJ559148, JN132891, JN133101 | 24 | 136.6307 |
| *Boophis* sp. 28 | Marojejy | ZSM 412/2005 (ZCMV 2062) | FJ559149, JN132890, JN133100 | 23 | RSA/RSB |
| *Boophis* sp. 33 | Andringitra | ZSM 731/2001 | AY848597, JN132894, JN133104 | 30 | no data |
| *Boophis sp. 35* | Ranomafana | ZSM 294/2006 (ZCMV 2976) | FJ559151, JN132888, JN133098 | 24 | 2223.354 |
| *Boophis* *spinophis* | Ranomafana (Ambatolahy) | ZSM 376/2004 (ZCMV 691) | AY848539, JN132883, JN133093 | 56.5 | 3925.027 |
| *Boophis* *tsilomaro* | Berara | MRSN A2000 | AJ314820, JN132898, JN133107 | 64 | 1391.149 |
| *Boophis tampoka* | Bemaraha | ZSM 96/2006 | AY848670, JN132920, JN133128 | 35 | 2409.668 |
| *Boophis tasymena* | Ranomafana (Maharira) | ZMA 20241 | AY848670, JN132921, DQ116476 | 23 | 5204.387 |
| *Boophis tephraeomystax* | Montagne d'Ambre | UADBA 24183 | AY848508, JN132922, JN133129 | 42 | 174961.8 |
| *Boophis ulftunni* | Ilampy (Masoala) | MRSN A4287  (FAZC 10396) | EU252143, JN132906, JN133114 | 24.2 | 74.52583 |
| *Boophis viridis* | Manombo | UADBA 24363 | AY848679, JN132923, JN133130, AY341676, AY341619, AY341805, EF100494 | 30 | 45994.86 |
| *Boophis vittatus* | Marojejy | ZSM 423/2005 (ZCMV 842) | FJ559158, JN132924, JN133131, AY341616, AY341673, AY341802, JN132827 | 25 | 558.9437 |
| *Boophis williamsi* | Ankaratra | ZSM 734/2001 | AY848624, JN132925, JN133132, JN132838, JN132841, JN132834, JN132828 | 37 | 1962.514 |
| *Boophis xerophilus* | Kirindy | ZFMK 66705 | AF215335, AF249069, -- | 39 | 6719.746 |
| *Gephyromantis ambohitra* | Montagne d'Ambre | ZSM 204/2004 | AY848309, JN132926, JN133133 | 37 | 385.0501 |
| *Gephyromantis asper* | Ranomafana | ZMA 20248 | AY848323, JN132928, JN133135, AY341598, AY341653, AY341783, JN132821 | 30 | 42367.93 |
| *Gephyromantis* *atsingy* | Bemaraha | ZSM 23/2006 | FJ559187, JN132934, JN133141 | no data | 2831.982 |
| *Gephyromantis azzurrae* | Isalo | MRSN A5311 | EF222302, JN132984, JN133206 | 43 | 2061.881 |
| *Gephyromantis blanci* | Andringitra | UADBA 20819 | AY848324, JN132929, JN133136 | 23 | 2906.507 |
| *Gephyromantis boulengeri* | Nosy Mangabe | ZSM 5057/2005  (ZCMV 2121) | DQ901391, JN132931, JN133138, DQ901389, DQ235450, DQ901396, JN132822 | 30 | 28332.24 |
| *Gephyromantis cornutus* | Vohidrazana | ZSM 164/2002 | FJ559164, JN132932, JN133139, EF100470, EF100477, EF100483, EF100495 | 40 | 12.42097 |
| *Gephyromantis corvus* | Isalo | ZFMK 70494 | AF215320, JN132933, JN133140 | 38 | RSA/RSB |
| *Gephyromantis decaryi* | Vondrozo | ZMA 20097 | AY848306, JN132935, JN133142 | 23 | 2558.72 |
| *Gephyromantis eiselti* | Ambavaniasy | ZSM 170/2005 | FJ559165, JN132936, JN133143 | 22 | 471.9969 |
| *Gephyromantis enki* | Ranomafana | UADBA (ZCMV 34) | AY848409, JN132937, DQ116486 | 21 | 571.3647 |
| *Gephyromantis granulatus* | Nosy Be | not preserved | AJ315927, JN132938, JN133144 | 45 | 20246.18 |
| *Gephyromantis horridus* | Tsaratanana | UADBA 10002 | FJ559167, JN132939, JN133145 | 28 | 5365.86 |
| *Gephyromantis klemmeri* | Marojejy | ZSM 278/2005 (FGZC 2727) | FJ559168, JN132941, JN133147, AY454363, AY454386, JN132835, JN132829 | 21 | RSA/RSB |
| *Gephyromantis leucocephalus* | Ste. Luce | not preserved | FJ559171, JN132942, JN133148 | 29 | 2558.72 |
| *Gephyromantis leucomaculatus* | Nosy Mangabe | ZSM 5079/2005  (ZCMV 882) | FJ559172, JN132943, JN133149 | 41 | 6024.171 |
| *Gephyromantis luteus* | Marojejy | ZSM 5088/2005  (ZCMV 2081) | FJ559173, JN132945, JN133151 | 43 | 65036.21 |
| *Gephyromantis malagasius* | Ambohitsara | ZMA 20247 | AY848353, JN132946, JN133152, EF100471, EF100478, EF100484, EF100496 | 23 | 5800.594 |
| *Gephyromantis moseri* | Andasibe | ZSM 93/2002 | FJ559174, JN132948, JN133154 | 40 | 24320.26 |
| *Gephyromantis plicifer* | Andohahela | ZSM 98/2004 | AY848419, JN132949, JN133155 | 48 | 695.5744 |
| *Gephyromantis pseudoasper* | Manongarivo | MVTIS 2000D61 | AY848421, JN132950, JN133156, DQ926891, DQ926892, DQ926893, EF100497 | 34 | 22407.43 |
| *Gephyromantis redimitus* | Nosy Mangabe | ZSM 5094/2005  (ZCMV 2150) | FJ559175, JN132951, JN133157, AY341648, AY341593, AY341778, EF100498 | 53 | 61719.81 |
| *Gephyromantis rivicola* | Andranofotsy | ZSM 180/2002 | DQ235449,DQ901386, JN133158, DQ235449, DQ901388, DQ901395, EF100500 | 24 | 2310.301 |
| *Gephyromantis runewsweeki* | Ranomafana (Maharira) | MNCN 42085 | AY848308, JN132952, JN133159 | 24 | RSA/RSB |
| *Gephyromantis salegy* | Ambolokopatrika | MRSN A2046 | AY848365, JN132953, JN133160 | 48 | 3925.027 |
| *Gephyromantis schilfi* | Marojejy | ZSM 268/2005  (FGZC 2735) | FJ559177, JN132954, JN133161 | 29 | 2397.248 |
| *Gephyromantis sculpturatus* | Vevembe | UADBA 24199 | FJ559178, JN132955, JN133162, AY341652, AY341597, AY341795, EF100499 | 43 | 12296.76 |
| *Gephyromantis silvanus* | Nosy Mangabe | ZSM 5095/2005  (ZCMV 888) | FJ559179, JN132956, JN133163, EF100472, EF100479, EF100485, EF100501 | 31 | 1651.989 |
| *Gephyromantis* sp. 5 | Andohahela | ZSM 182/2005  (FGZC 2459) | FJ559182, JN132930, JN133137 | no data | RSA/RSB |
| *Gephyromantis* sp. 11 | Marojejy | ZSM 222/2005 (FGZC 2843) | FJ559188, JN132940, JN133146 | 23.5 | RSA/RSB |
| *Gephyromantis* sp. 13 | Ranomafana | ZMA 19421 | EF100471, JN132947, JN133153 | 23 | 211.1565 |
| *Gephyromantis* sp. 17 | Marojejy | ZSM 5081/2005  (ZCMV 2086) | FJ559191, JN132944, JN133150 | 41 | 372.6291 |
| *Gephyromantis* sp. 18 | Ambolokopatrika | FAZC 7230 | FJ559163, ---, --- | no data | no data |
| *Gephyromantis spinifer* | Midongy du Sud | UADBA 25495 | FJ559198, JN132957, JN133164 | 35 | 7874.896 |
| *Gephyromantis striatus* | Marojejy | UADBA? (FGZC 2774) | AY341650, JN132958, JN133165 | 24 | 1627.147 |
| *Gephyromantis* *tahotra* | Marojejy | UADBA (ZCMV 2016) | FJ559180, JN132927, JN133134 | 36 | RSA/RSB |
| *Gephyromantis tandroka* | Marojejy | ZSM 322/2005  (FGZC 2816) | FJ559199, JN132959, JN133166 | 39 | RSA/RSB |
| *Gephyromantis thelenae* | Mantadia | UADBA (FGMV 2001.1014) | AY848331, JN132960, JN133167 | 23 | 24.84194 |
| *Gephyromantis tschenki* | Ranomafana | ZSM 651/2003 / ZMA 19392 | AY848377, JN132961, JN133168 | 36 | 658.3115 |
| *Gephyromantis ventrimaculatus* | Ranomafana (Ranomafanakely) | ZSM 537/2006  (ZCMV 3362) | FJ559200, JN132962, JN133169 | 25 | 17525.99 |
| *Gephyromantis webbi* | Nosy Mangabe | ZSM 5099/2005  (ZCMV 872) | FJ559201, JN132963, JN133170 | 25 | RSA/RSB |
| *Gephyromantis zavona* | Tsaratanana | ZSM 627/2001 | AJ314795, JN132964, JN133171 | 41 | 248.4194 |
| *Guibemantis albolineatus* | Andohahela | ZSM 171/2005  (FGZC 2432) | FJ559202, JN132965, JN133172 | 24 | 3925.027 |
| *Guibemantis* *annulatus* | Ste. Luce | RML 290 | EF472520 , JN132980, JN133186 | 25 | RSA/RSB |
| *Guibemantis bicalcaratus* | Nosy Boraha | ZSM 429/2006  (ZCMV 3244) | FJ559203, GU983156, GU983142, EF100473, AY341581, AY341767, EF100502 | 25 | 1664.41 |
| *Guibemantis depressiceps* | Ranomafana (Vohiparara) | ZMA 20238 | AY848066, JN132973, JN133179, AY341645, AY341590, AY341775, EF100503 | 45 | 10831.09 |
| *Guibemantis flavobrunneus* | Mangoro river region | ZSM 260/2005  (FGZC 2655) | EF472510, JN132975, JN133181 | 33 | RSA/RSB |
| *Guibemantis kathrinae* | An'Ala | MVTIS GA2002 | AY684189, JN132977, JN133183 | 59 | 3925.027 |
| *Guibemantis liber* | Mandraka | UADBA 20601 | AY848084, GU983157, GU983143, AY341644, AY341589, AY341774, EF100504 | 29 | 204598.2 |
| *Guibemantis pulcher* | Ranomafana | ZMA 20242 | AY848096, JN132979, JN133185 | 25 | 67309.24 |
| *Guibemantis punctatus* | Ambohitantely | UADBA? (ZCMV 5501) | FJ559205, JN132981, JN133187 | 25 | 981.2568 |
| *Guibemantis* sp. 3 | Andasibe | ZSM 250/2002 | AY454376, JN132966, JN133173 | 24 | RSA/RSB |
| *Guibemantis* sp. 5 | Vevembe | ZMA 20115 | FJ559207, JN132978, JN133184 | 29 | 3154.927 |
| *Guibemantis* sp. 8 | Besariaka | UADBA (ZCMV 466) | AY848035, JN132972, -- | 25 | RSA/RSB |
| *Guibemantis* sp. 9 | Nosy Boraha | ZSM 425/2006  (ZCMV 3240) | FJ559208, JN132967, JN133174 | 25 | 298.1033 |
| *Guibemantis* sp. 10 | Manombo | ZMA 20119 | AY848076, JN132976, JN133182 | 33 | 7315.952 |
| *Guibemantis* sp. 12 | Manongarivo | ZMA 19582 | FJ559209, JN132970, JN133177 | 25 | RSA/RSB |
| *Guibemantis* sp. 14 | Fierenana | ZMA 19347 | FJ559210, JN132971, JN133178 | 25 | RSA/RSB |
| *Guibemantis* sp. 15 | Marojejy | ZSM 5063/2005  (ZCMV 2044) | EF472505, ---, --- | 25 | RSA/RSB |
| *Guibemantis* sp. 17 | Marojejy | ZSM 5062/2005  (ZCMV 2011) | FJ559211, JN132969, JN133176 | 25 | RSA/RSB |
| *Guibemantis* sp. 19 | Andohahela | ZSM 319/2005  (FGZC 2434) | EF488668, JN132974, JN133180 | no data | RSA/RSB |
| *Guibemantis* sp. 20 | Manombo | UADBA?  ZCMV 5496 | FJ559212, JN133051, JN133275 | no data | RSA/RSB |
| *Guibemantis timidus* | Toamasina | ZMA 19492 | AY684185, JN132982, JN133188 | 55 | 25810.78 |
| *Guibemantis tornieri* | Mantadia | UADBA (FGMV 2001 1094) | FJ559214, JN132983, JN133189 | 51 | 21637.33 |
| *Guibemantis* *wattersoni* | Ste Luce | ZSM 176/2005  (FGZC 2588) | EF472508, JN132968, JN133175 | 25 | 981.2568 |
| *Laliostoma labrosum* | Bemaraha | ZSM 16/2006 | FJ559215, JN132849, JN133058, AY341679, AF249010, AF249106, DQ019530 | 48 | 433864.5 |
| *Mantella aurantiaca* | Torotorofotsy | ZCMV 972 | FJ559216, AY723566, JN133190 | 24 | 62.10486 |
| *Mantella baroni* | Ranomena | ZSM 361/2006  (ZCMV 3343) | FJ559217, DQ889429, JN133191 | 30 | 28754.55 |
| *Mantella bernhardi* | Vevembe | ZMA 20190 | FJ559218, DQ278731, JN133192 | 19 | 7651.318 |
| *Mantella betsileo* | Isalo | MVTIS AC155 | FJ559219, AY263295, JN133193 | 21 | 98709.46 |
| *Mantella cowani* | Antoetra | MVTIS KCW112 | FJ559220, AY263301, JN133194 | 29 | 18345.78 |
| *Mantella crocea* | Ampangadimbolana | MVTIS CS61 | AJ438897, AY723623, JN133195 | 24 | 6334.695 |
| *Mantella ebenaui* | Nosy Be | UADBA (FGMV 2001.6) | FJ559221, EF179691, JN133196, AJ438886, AF215174, AY263280, EF100505 | 21 | 88921.74 |
| *Mantella expectata* | pet trade | MVTIS CS06 | AJ438889, AY263299, JN133197 | 26 | 2061.881 |
| *Mantella haraldmeieri* | Manatantely | ZSM 156/2004 | FJ559222, AY263302, JN133199 | 27 | 2521.457 |
| *Mantella laevigata* | Marojejy | UADBA?  FGZC 2760 | FJ559223, GU983155, GU983141 | 29 | 10657.19 |
| *Mantella madagascariensis* | Ranomafana | ZSM 670/2003 | FJ559224, EF100464, JN133200, AJ438892, AF249005, AF249101, DQ019532 | 22 | 1378.728 |
| *Mantella manery* | Marojejy | ZSM 5234/2005 | FJ559225, EF179704, JN133201 | 29 | RSA/RSB |
| *Mantella milotympanum* | Fierenana | ZMA 19317 | FJ559226, AY723662, JN133202 | 23 | 37.26291 |
| *Mantella nigricans* | Marojejy | ZSM 450/2005  (ZCMV 846) | FJ559227, DQ889356, JN133203 | 28 | 15066.64 |
| *Mantella pulchra* | An'Ala | ZSM 372/2006  (ZCMV 2490) | FJ559228, AY723693, JN133204 | 23 | 13799.7 |
| *Mantella* sp. 1 | Tranomaro | UADBA?  (FGZC 2325) | FJ559229, EF179619, JN133198 | 23 | 6719.746 |
| *Mantella viridis* | pet trade | MVTIS CS05 | FJ559230, JN132986, JN133205 | 25 | 86.9468 |
| *Mantidactylus aerumnalis* | An'Ala | MVTIS 2002A2 | AY848125, JN132986, JN133208 | 27 | 18581.77 |
| *Mantidactylus albofrenatus* | Andasibe | UADBA (FGMV 2001.1169) | AY848267, JN132987, JN133209 | 23 | 2260.617 |
| *Mantidactylus alutus* | Mantasoa | ZSM 355/2000 | FJ559232, JN132988, JN133210 | 26 | 35946.29 |
| *Mantidactylus ambreensis* | Montagne d'Ambre | ZSM 229/2004 | AY848129, JN132989, JN133211 | 38 | 5465.227 |
| *Mantidactylus argenteus* | Mantadia | ZSM 47/2002 | DQ235447, JN132990, JN133212, DQ235454, DQ235447, DQ235444, JN132823 | 27 | 56602.37 |
| *Mantidactylus bellyi* | Montagne d'Ambre | MVTIS E179 | FJ559233, JN132991, JN133213 | 41 | 4943.547 |
| *Mantidactylus betsileanus* | Andasibe | ZSM 391/2006  (ZCMV 3260) | FJ559234, JN132992, JN133214 | 28 | 10272.14 |
| *Mantidactylus biporus* | Moramanga | ZSM 398/2006  (ZCMV 3259) | FJ559235, JN132999, JN133221 | 27 | 3925.027 |
| *Mantidactylus bourgati* | Andringitra | MVTIS_E53_9 | AY848237, JN133006, JN133228 | 40 | RSA/RSB |
| *Mantidactylus brevipalmatus* | Antoetra | UADBA 21181 | AY848132, JN133007, JN133229 | 35 | 33946.52 |
| *Mantidactylus charlotteae* | Nosy Mangabe | ZSM 5070/2005  (ZCMV 2115) | FJ559236, JN133008, JN133230, AY341661, AY341605, AY341790, EF100507 | 26 | 15799.48 |
| *Mantidactylus cowanii* | Mantadia | ZSM 171/2002 | AY341711, DQ235429, JN133232 | 40 | 18233.99 |
| *Mantidactylus curtus* | Col de Tapias (Antoetra) | UADBA (ZCMV 10) | AY848288, JN133011, JN133234 | 38.4 | 2658.088 |
| *Mantidactylus delormei* | Andringitra | ZSM 751/2001 | AY848148, JN133015, JN133238 | no data | 74.52583 |
| *Mantidactylus femoralis* | Isalo | ZMA 19469 | AY848138, JN133016, JN133239, AY341658, AY341602, AY341787, EF100509 | 37 | no data |
| *Mantidactylus grandidieri* | Ambohitsara | ZMA 20216 | AY848168, JN133019, JN133242, AY341660, AY341604, AY341789, EF100506 | 110 | 19351.87 |
| *Mantidactylus guttulatus* | Tsaratanana | ZSM 644/2001 | FJ559237, JN133020, JN133243 | 110 | 3577.24 |
| *Mantidactylus lugubris* | Andasibe | ZSM 166/2002 | AY848178, JN133022, JN133245, AY341656, AY341600, AY341785, EF100508 | 35 | 2819.561 |
| *Mantidactylus madecassus* | Andringitra | UADBA 20639 | AY848297, JN133026, JN133249 | 30 | RSA/RSB |
| *Mantidactylus majori* | Ranomafana | UADBA?  (ZCMV 252) | AY848187, JN133027, JN133250 | 47 | 9067.309 |
| *Mantidactylus melanopleura* | Andasibe | UADBA (FGMV 1276) | FJ559239, JN133029, DQ116491 | 40 | 106149.6 |
| *Mantidactylus noralottae* | Isalo | MRSN 5254 | EF222308, JN132985, JN133207 | no data | RSA/RSB |
| *Mantidactylus opiparis* | Ambohitantely | ZSM 5050/2005  (FGZC 2125) | FJ559240, JN133033, JN133255 | 26 | 171123.7 |
| *Mantidactylus pauliani* | Ankaratra | ZSM 756/2001 | AY848298, JN133034, JN133256 | 32 | 173.8936 |
| *Mantidactylus* sp. 7 | Manantantely | ZSM 146/2004 | AY848141, JN133035, JN133257 | 17.5 | 3179.769 |
| *Mantidactylus* sp. 13 | Ranomafana | ZSM 748/2003 | FJ559248, JN133009, JN133231 | 26 | 6632.799 |
| *Mantidactylus* sp. 14 | Isalo | ZSM 23/2004 | AY848286, JN133038, JN133260 | 36 | 4682.706 |
| *Mantidactylus* sp. 17 | Ambohitantely | ZSM 180/2005  (FGZC 2146) | FJ559251, JN133004, JN133226 | 25 | RSA/RSB |
| *Mantidactylus* sp. 18 | Ambohitantely | ZSM 219/2005  (FGZC 2143) | FJ559252, JN133013, JN133236 | 40 | RSA/RSB |
| *Mantidactylus* sp. 19 | Ankaratra | FGMV 2002.2109 | AY848254, JN133012, JN133235 | 40 | 12.42097 |
| *Mantidactylus* sp. 20 | Itremo | ZSM 763/2001 | AY848219, ---, --- | 35 | RSA/RSB |
| *Mantidactylus* sp. 22 | Andranofotsy | ZSM 122/2002 | FJ559253, JN133003, JN133225 | 23 | RSA/RSB |
| *Mantidactylus* sp. 23 | Andasibe | ZSM 85/2002 | FJ559254, JN133002, JN133224 | 23 | RSA/RSB |
| *Mantidactylus* sp. 24 | Ranomafana | ZSM 434/2006  (ZCMV 3366) | FJ559255, JN133005, JN133227 | 23 | RSA/RSB |
| *Mantidactylus* sp. 26 | Andranofotsy | ZSM 176/2002 | FJ559257, JN132995, JN133217 | 33 | 3328.82 |
| *Mantidactylus* sp. 27 | Nosy Boraha | ZSM 423/2006  (ZCMV 3390) | FJ559258, JN132998, JN133220 | 25 | 335.3662 |
| *Mantidactylus* sp. 28 | Andohahela | ZSM 91/2004 | AY848274, JN132993, JN133215 | 24.3 | 21637.33 |
| *Mantidactylus* sp. 29 | Tolagnaro | ZSM 70/2004 | AY848273, JN132997, JN133219 | 26 | 86.9468 |
| *Mantidactylus* sp. 30 | Andohahela | ZSM 205/2005  (FGZC 2376) | FJ559259, JN133014, JN133237 | 20 | RSA/RSB |
| *Mantidactylus* sp. 32 | Manongarivo | UADBA (FGMV 2002.756) | AY848263, JN133000, JN133222 | 22 | 3664.187 |
| *Mantidactylus* sp. 33 | Tsaratanana (Antsahamanara campsite) | UADBA (MV 2001.60) | FJ559261, JN133001, JN133223 | 20 | 3751.133 |
| *Mantidactylus* sp. 34 | Vohidrazana | UADBA (FGMV 2001.1156) | FJ559262, JN132994, JN133216 | 25 | RSA/RSB |
| *Mantidactylus* sp. 36 | Maroantsetra | ZSM 454/2005  (ZCMV 803) | AY848259, JN132996, JN133218 | 32 | 2558.72 |
| *Mantidactylus* sp. 36 | Toamasina | UADBA  (ZCMV 805) | AY848260, ---, --- | 32 | 2558.72 |
| *Mantidactylus* sp. 38 | Andasibe | MV 2001.1277 | DQ235430, ---, --- | 45 | no data |
| *Mantidactylus* sp. 40 | Manongarivo | ZSM 855/2003 | FJ559265, JN133018, JN133241 | 40 | no data |
| *Mantidactylus* sp. 41 | Andapa | ZSM 221/2005  (FGZC 2719) | FJ559266, JN133028, JN133251 | 40 | 2956.191 |
| *Mantidactylus* sp. 42 | Montagne d'Ambre | UADBA (FGMV 2002.929) | FJ559267, ---, --- | 40 | no data |
| *Mantidactylus* sp. 43 | Marojejy | ZSM 253/2005  (FGZC 2797) | FJ559268, ---, --- | 40 | no data |
| *Mantidactylus* sp. 44 | Ambohitsara | UADBA?  (ZCMV 5865) | FJ559269, ---, ---- | 45 | no data |
| *Mantidactylus* sp. 45 | Manongarivo (Tsaratanana) | ZSM 854/2003 | FJ559270, JN133030, JN133252 | 45 | no data |
| *Mantidactylus* sp. 46 | Marojejy | ZSM 5089/2005  (ZCMV 2024) | FJ559271, JN133031, JN133253 | 45 | no data |
| *Mantidactylus* sp. 47 | Ambatolahy | ZSM 442/2006  (ZCMV 2860) | FJ559272, JN133032, JN133254 | no data | no data |
| *Mantidactylus* sp. 48 | Itremo | ZSM 745/2001 | FJ559273, JN133010, JN133233 | 35 | 10644.77 |
| *Mantidactylus* sp. 49 | Andohahela | ZSM 69/2004 | AY848174, JN133024, JN133247 | 35 | 37.26291 |
| *Mantidactylus* sp. 52 | Marojejy | ZSM 305/2005  (FGZC 2767) | FJ559274, JN133023, JN133246 | 35 | RSA/RSB |
| *Mantidactylus* sp. 53 | Masoala | FAZC 5132 | AY848173, ---, --- | 35 | 1925.251 |
| *Mantidactylus* sp. 54 | Besariaka | FAZC 7609 | AY848180, JN133025, JN133248 | 35 | 2148.828 |
| *Mantidactylus* sp. 57 | Nosy Mangabe | ZSM 5076/2005  (ZCMV 2158) | FJ559277, JN133021, JN133244 | 110 | 19351.87 |
| *Mantidactylus tricinctus* | Manombo | ZMA 20100 | AY848128, JN133036, JN133258 | 20 | 3179.769 |
| *Mantidactylus* sp. aff. *tricinctus* | An'Ala | ZSM 375/2006  (ZCMV 1404) | FJ559281, ---, --- | 20 | 2074.302 |
| *Mantidactylus ulcerosus* | Ankarafantsika | UADBA?  (FGMV 2001.403) | FJ559282, JN133037, JN133259, AY341654, AF249006, AF249102, EF100510 | 34 | 65930.52 |
| *Mantidactylus zipperi* | An'Ala | ZSM 538/2006  (ZCMV 1403) | FJ559283, JN133039, JN133261 | 23 | 372.6291 |
| *Mantidactylus zolitschka* | An'Ala | ZSM 184/2003 | AY324811, JN133040, JN133262 | 31 | RSA/RSB |
| *Spinomantis aglavei* | Maharira | UADBA (ZCMV 240) | AY848394, GU983158, GU983144, EF100474, EF100480, EF100486, EF100511 | 48 | 75643.72 |
| *Spinomantis bertini* | Andohahela | ZSM 173/2005  (FGZC 2431) | FJ559301, JN133041, JN133263, JN132839, JN132842, JN132836, JN132830 | 23 | 3887.764 |
| *Spinomantis elegans* | Andringitra | ZSM 971/2003 | AY848405, EF100467, JN133266, EF100475, EF100481, EF100487, EF100512 | 60 | 10023.72 |
| *Spinomantis fimbriatus* | Andasibe | MVTIS Tad417-2004 | FJ559302, JN133044, JN133267 | 39 | 27835.4 |
| *Spinomantis guibei* | Andohahela | ZSM 269/2005  (FGZC 2435) | FJ559304, JN133045, JN133268, JN132840, JN132843, JN132837, JN132831 | 35 | 385.0501 |
| *Spinomantis massi* | Manongarivo | UADBA (FGMV 2002.803) | AY848412, JN133046, JN133269 | 37 | 645.8905 |
| *Spinomantis microtis* | Andohahela | ZSM 5212/2005  (FGZC 2532) | FJ559305, JN133047, JN133270 | 48 | 360.2082 |
| *Spinomantis peraccae* | Maharira | ZMA 20251 | AY848416, JN133048, JN133271 | 44 | 82798.2 |
| *Spinomantis phantasticus* | Vohidrazana | ZMA 19627 | AY848417, JN133049, JN133272 | 38 | 2409.668 |
| *Spinomantis* sp. 6 | Andohahela (low altitude) | UADBA (FGZC 148) | AY848396, JN133042, JN133264 | 23 | RSA/RSB |
| *Spinomantis* sp. 7 | Ranomafana | ZSM 806/2003 | AY848400, JN133043, JN133265 | 23 | RSA/RSB |
| *Spinomantis* sp. 8 | Maharira | ZMA 20044 | AY848382, GU983159, GU983145 | 25 | 2707.772 |
| *Tsingymantis antitra* | Ankarana | UADBA?  (FGZC 531) | AY848213, JN133050, JN133273, DQ901387, DQ901392, DQ901393, JN132832 | 54 | 2123.986 |
| *Wakea madinika* | Antsirasira | -- | AY341703, DQ235419, JN133274, AY341587, AY341642, AY341772, JN132833 | 13 | RSA/RSB |
| *Polypedates* spp. (composite) | --- | --- | AF215358, AF249089, AY458598, AF249028, AY341685, AY341810, AY323802 | -- | -- |

Supplementary Table S2. Primer information. If more than one forward – and reverse primer are given, different combinations were used for amplification.

| **Gene fragment** | **forward primer** | **reverse primer** |
| --- | --- | --- |
| 12S | 12SAL  5’-AAACTGGGATTAGATACCCCACTAT-3’ | 12SBH  5’-GAGGGTGACGGGCGGTGTGT-3’ |
| 16S1 | 16SAL  5’-CGCCTGTTTATCAAAAACAT-3’ | 16SBH 5’-CCGGTCTGAACTCAGATCACGT-3’ |
| 16S2 | 16SL3  5’-AGCAAAGAHYWWACCTCGTACCTTTTGCAT-3’ | 16SAH  5’-ATGTTTTTGATAAACAGGCG-3’ |
| cob | CBJ10933  5’-TATGTTCTACCATGAGGACAAATATC-3’  MVZ15L-mod  5’-AACTWATGGCCCMCACMATMCGWAA-3’  Cytb-a  5’-CCATGAGGACAAATATCATTYTGRGG-3’ | Cytb-c,  5’-CTACTGGTTGTCCTCCGATTCATGT-3’  CytbAR-H-mod  5’-TAWARGGRTCYTCKACTGGTTG-3’ |
| cox1 | Vert-F1  5’-TTCTCAACCAACCACAAAGACATTGG-3’  Vert-F2  5’-TCAACCAACCACAAAGACATTGGCAC-3’  LCO1490  5’-GGTCAACAAATCATAAAGATATTGG-3’ | Vert-R1  5’-TAGACTTCTGGGTGGCCAAAGAATCA-3’  HCO2198  5’-TAAACTTCAGGGTGACCAAAAAATCA-3’ |
| rhodopsin exon 1 | Rhod-ma  5’- AACGGAACAGAAGGYCC-3’ | Rhod-md  5’- GTAGCGAAGAARCCTTC-3’ |
| RAG2 (first PCR) | 31FN.Venk  5’-TTYGGICARAARGGITGGCC-3’ | Rag2Lung.460R  5’-GCATYGRGCATGGACCCARTGICC-3’ |
| RAG2 (second PCR) | Rag2A.F35,  5’- TGG CCI AAA MGI TCY TGY CCM ACW  GG-3’  Rag2.Lung.35F  5’-GGCCAAAGAGRTCYTGTCCIACTGG-3’ | Rag2.Lung.320R  5’-AYCACCCATATYRCTACCAAACC-3’ |

Supplementary Table S3**.** Best-fit models of evolution for all-taxa and reduced-taxa datasets and partitions as selected by MrModeltest 2.2. –'X' refers to codon position(s).

| **All-taxa dataset** | | **Reduced-taxa dataset** | |
| --- | --- | --- | --- |
| **Partition** | **Model** | **Partition** | **Model** |
| 16SrRNA | GTR+I+G | rRNA | GTR+I+G |
| cob | GTR+I+G | cob–1 | GTR+I+G |
| cob–1 | GTR+I+G | cob–2 | GTR+G |
| cob–2 | GTR+I+G | cob–3 | SYM+I+G |
| cob–12 | GTR+I+G | rhod–1 | K80+G |
| cob–3 | GTR+I+G | rhod–2 | F81+G |
| cox1 | GTR+I+G | rhod–3 | HKY+G |
| cox1–1 | GTR+I+G | rag2–1 | GTR+I+G |
| cox1–2 | HKY+I | rag2–2 | GTR+I+G |
| cox1–12 | HKY+I+G | rag2–3 | GTR+G |
| cox1–3 | GTR+I+G | cox1–1 | GTR+I+G |
| cob–cox1–12 | GTR+I+G | cox1–2 | GTR+I |
| cob–cox1–3 | GTR+I+G | cox1–3 | GTR+G |
| unpartitioned dataset 16S, cob, cox1 | GTR+I+G | unpartitioned dataset rRNA, cob, rhod, rag2, cox1 | GTR+I+G |

Supplementary Table S4. Calibration points for ultrametric tree computation. Primary calibrations (Comoros frogs) are taken from Vences et al. (2003). Secondary calibrations follow Kurabayashi et al. (2008). The root age was not fixed. Calibration types are fixage = node has fixed age, or minage – maxage: node age is given as interval (= confidence intervals taken as secondary calibration from Kurabayashi et al., 2007).

| **Node** | **Taxon 1** | **Taxon 2** | **Calibration type / date [Mya]** |
| --- | --- | --- | --- |
| Colonization of volcanic Comoro island | *Blommersia* sp. Comoros | *Blommersia wittei* | fixage=8.7 |
| Colonization of volcanic Comoro island | *Boophis* sp. Comoros | *Boophis doulioti* | fixage=8.7 |
| Split between *Wakea* and *Mantella* | *Wakea madinika* | *Mantella betsileo* | minage=13.9 maxage=23.3 |
| Split between *Wakea/Mantella* and *Blommersia* | *Wakea madinika* | *Blommersia blommersae* | minage=19.8 maxage=30.0 |
| Split between *Wakea/Mantella/Blommersia* and *Guibemantis* | *Blommersia blommersae* | *Guibemantis albolineatus* | minage=14.6 maxage=24.2 |
| Split between *Spinomantis* and pond breeding lineage | *Guibemantis albolineatus* | *Spinomantis aglavei* | minage=27.5 maxage=38.8 |
| Split between pond breeders + *Spinomantis* and stream breeding + endotrophic lineage in the Mantellinae | *Spinomantis aglavei* | *Boehmantis microtympanum* | minage=29.7 maxage=41.1 |
| Split between *Mantidactylus* and *Gephyromantis* | *Mantidactylus guttulatus* North | *Gephyromantis thelenae* | minage=22.4 maxage=33.5 |
| Split between *Mantidactylus/Gephyromantis* and *Boehmantis* | *Gephyromantis cornutus* | *Boehmantis microtympanum* | minage=25.4 maxage=36.8 |
| Split between *Laliostoma* and *Aglyptodactylus* | *Laliostoma labrosum* | *Aglyptodactylus laticeps* | minage=29.2 maxage=41.4 |

Supplementary Table S5. Factor loadings for 21 climatic variables. Varimax-rotated coordinate system, marked loadings >0.7.

| bio^a^ | **PC1** | **PC2** | **PC3** | **PC4** |
| --- | --- | --- | --- | --- |
| wb | **0.961** | -0.012 | 0.118 | 0.164 |
| etp | -0.162 | 0.694 | 0.679 | 0.057 |
| 1 | **0.986** | 0.059 | 0.100 | 0.113 |
| 2 | -0.226 | -0.694 | -0.247 | -0.522 |
| 3 | 0.029 | -0.695 | 0.012 | 0.403 |
| 4 | **0.967** | 0.015 | 0.132 | 0.215 |
| 5 | 0.241 | 0.653 | 0.691 | 0.122 |
| 6 | 0.297 | -0.002 | **0.928** | 0.114 |
| 7 | 0.120 | **0.951** | 0.201 | 0.025 |
| 8 | -0.029 | **-0.959** | 0.041 | -0.056 |
| 9 | 0.246 | 0.165 | **0.941** | 0.113 |
| 10 | 0.155 | **0.941** | 0.225 | 0.034 |
| 11 | -0.088 | 0.447 | **0.810** | 0.148 |
| 12 | 0.212 | **0.920** | 0.265 | 0.050 |
| 13 | -0.526 | 0.377 | -0.225 | -0.686 |
| 14 | **0.985** | -0.044 | 0.026 | -0.124 |
| 15 | **0.881** | 0.250 | 0.191 | 0.346 |
| 16 | -0.273 | -0.488 | -0.297 | **-0.756** |
| 17 | **0.977** | 0.179 | 0.050 | 0.017 |
| 18 | **0.949** | 0.177 | 0.150 | 0.174 |
| 19 | **0.981** | 0.169 | 0.063 | 0.014 |
| **Expl.Var** | **8.123** | **6.197** | **3.811** | **1.804** |
| **Prp.Totl** | **38.68%** | **29.51%** | **18.15%** | **8.59%** |

a) Explanations for the 'bio' variables can be found in Hijmans et al. (2005) and for 'wb' and 'etp' in Kremen et al. (2008).

Supplementary Table S6. Eigenvalues of PCA for climatic variables in Varimax-raw rotated coordinate system.

| **Principal Component** | **Eigenvalue** | **% Total variance** | **Cumulative Eigenvalue** | **Cumulative %** |
| --- | --- | --- | --- | --- |
| **1** | 10.44 | 49.71 | 10.44 | 49.71 |
| **2** | 5.94 | 28.27 | 16.38 | 77.98 |
| **3** | 2.42 | 11.53 | 18.80 | 89.51 |
| **4** | 1.14 | 5.42 | 19.93 | 94.93 |

Supplementary Table S7. Univariate tests of significance for factorial regression analysis through the origin to determine the effect of age, spatial distance and bioclimatic distance, and their interaction terms on standardized a - body size and b, c - range size contrasts in sister species range size estimators using large (RSA) and small (RSB) buffer zones for one- or two locality species. Significant effects in bold. Centroid distance was the best predictor for SVL contrasts, while range size contrasts were best predicted by bioclimatic distance and its interaction terms for both RSA and RSB

| a - Univariate tests of significance for standardized contrasts SVL | | | | |
| --- | --- | --- | --- | --- |
|  | **SS** | **MS** | **F** | **p** |
| Cen distance | 486.875 | 486.8749 | 7.218207 | **0.010218** |
| Age [Mya] | 178.197 | 178.1975 | 2.641882 | 0.111388 |
| Bioclim Dist PC1-PC4 | 25.087 | 25.0873 | 0.371934 | 0.545161 |
| Cen distance*Age [Mya] | 137.282 | 137.2815 | 2.035279 | 0.160907 |
| Cen distance*Bioclim Dist PC1-PC4 | 20.150 | 20.1497 | 0.298731 | 0.587504 |
| Age [Mya]*Bioclim Dist PC1-PC4 | 35.921 | 35.9212 | 0.532553 | 0.469493 |
| Cen distance*Age [Mya]*Bioclim Dist PC1-PC4 | 9.345 | 9.3454 | 0.138551 | 0.711554 |
| Error | 2900.391 | 67.4510 |  |  |
| b - Univariate tests of significance for standardized contrasts RSA | | | | |
|  | **SS** | **MS** | **F** | **p** |
| Cen distance | 0.320 | 0.320 | 0.00899 | 0.924881 |
| Age [Mya] | 4.055 | 4.055 | 0.11414 | 0.737119 |
| Bioclim Dist PC1-PC4 | 1139.471 | 1139.471 | 32.07681 | **0.000001** |
| Cen distance*Age [Mya] | 0.269 | 0.269 | 0.00756 | 0.931110 |
| Cen distance*Bioclim Dist PC1-PC4 | 430.007 | 430.007 | 12.10496 | **0.001165** |
| Age [Mya]*Bioclim Dist PC1-PC4 | 729.853 | 729.853 | 20.54581 | **0.000046** |
| Cen distance*Age [Mya]*Bioclim Dist PC1-PC4 | 309.120 | 309.120 | 8.70191 | **0.005126** |
| Error | 1527.497 | 35.523 |  |  |
| c - Univariate tests of significance for standardized contrasts RSB | | | | |
|  | **SS** | **MS** | **F** | **p** |
| Cen distance | 1.647 | 1.647 | 0.04647 | 0.830339 |
| Age [Mya] | 4.612 | 4.612 | 0.13013 | 0.720059 |
| Bioclim Dist PC1-PC4 | 1131.341 | 1131.341 | 31.92346 | **0.000001** |
| Cen distance*Age [Mya] | 0.002 | 0.002 | 0.00007 | 0.993503 |
| Cen distance*Bioclim Dist PC1-PC4 | 433.300 | 433.300 | 12.22658 | **0.001107** |
| Age [Mya]*Bioclim Dist PC1-PC4 | 725.764 | 725.764 | 20.47916 | **0.000047** |
| Cen distance*Age [Mya]*Bioclim Dist PC1-PC4 | 311.631 | 311.631 | 8.79340 | **0.004917** |
| Error | 1523.884 | 35.439 |  |  |

Supplementary Table S8**.** Results of multiple regressions between substitution rate, body size and range size performed with CoEvol.

a – multiple regression with the small range size estimate

|  |  | **Ds** | **SVL** | **RSB (0.001)** |
| --- | --- | --- | --- | --- |
| Ds | cov  r^2^  pp | 0.305  1  -- | -0.0337  0.0307  **0.014** | 0.197  0.00988  **0.85** |
| SVL | cov  r^2^  pp |  | 0.122  1  -- | 0.16  0.0162  **0.98** |
| RSB | cov  r^2^  pp |  |  | 13  1  -- |

b – multiple regression with the large range size estimate

|  |  | **Ds** | **SVL** | **RSA**  **(0.0158)** |
| --- | --- | --- | --- | --- |
| Ds | cov  r^2^  pp | 0.303  1  -- | -0.0361  0.0362  **0.0072** | 0.0158  0.000107  0.52 |
| SVL | cov  r^2^  pp |  | 0.12  1  -- | -0.00783  8.47e-005  0.43 |
| RSA | cov  r^2^  pp |  |  | 6.22  1  -- |
|  |  |  |  |  |

Supplementary Figures

Supplementary Figure S1. Topology obtained for the reduced-taxa dataset (containing 3760 bp for fragments of 12S, 16S, cob, cox1, rhodopsin 1, and RAG2 genes) as found by Bayesian tree searches under maximum partitioning strategy (20,000,000 generations), heuristic ML searches (first numbers), and MP searches (second numbers, if present). The topology did not differ. Posterior probabilities * (95-98), ** (99-100) are depicted below nodes, ML bootstrap values (performed using the rapid bootstrapping algorithm with estimated number of bootstrap replicates) are numbers above branches.


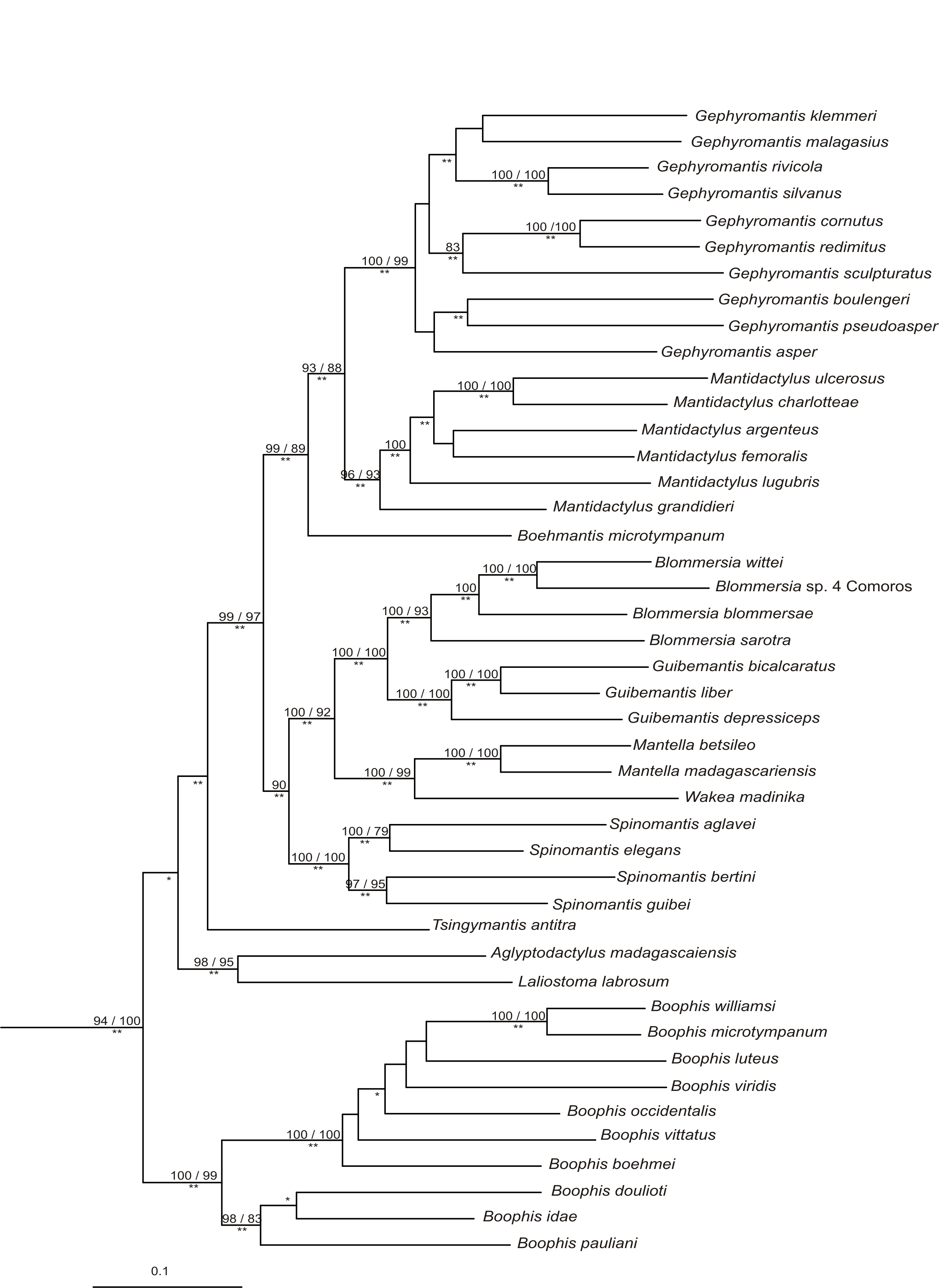


Supplementary Figure S2. (I-III) - Phylogeny of mantellids based on DNA sequences of three mitochondrial genes. The tree is a majority-rule consensus tree from a Bayesian analysis (30 million generations) of the all-taxa data set, with a number of deep nodes (indicated by light blue circles) constrained following a previous multi-gene analysis of a reduced taxon set (see Supplementary Figure S1). Bayesian posterior probabilities are indicated by * (95-98) or ** (99-100). The subgenera *Mantidactylus* (*Ochthomantis*) and *Guibemantis* (*Pandanusicola*) were excluded from analysis due to severe uncertainties in taxonomy as well as the two undescribed Comoroan species (gray taxa, see also Supplementary Material S1). Dark green shade indicates analyzed sister species with sufficiently high support values. *Polypedates* was used as outgroup (not shown).


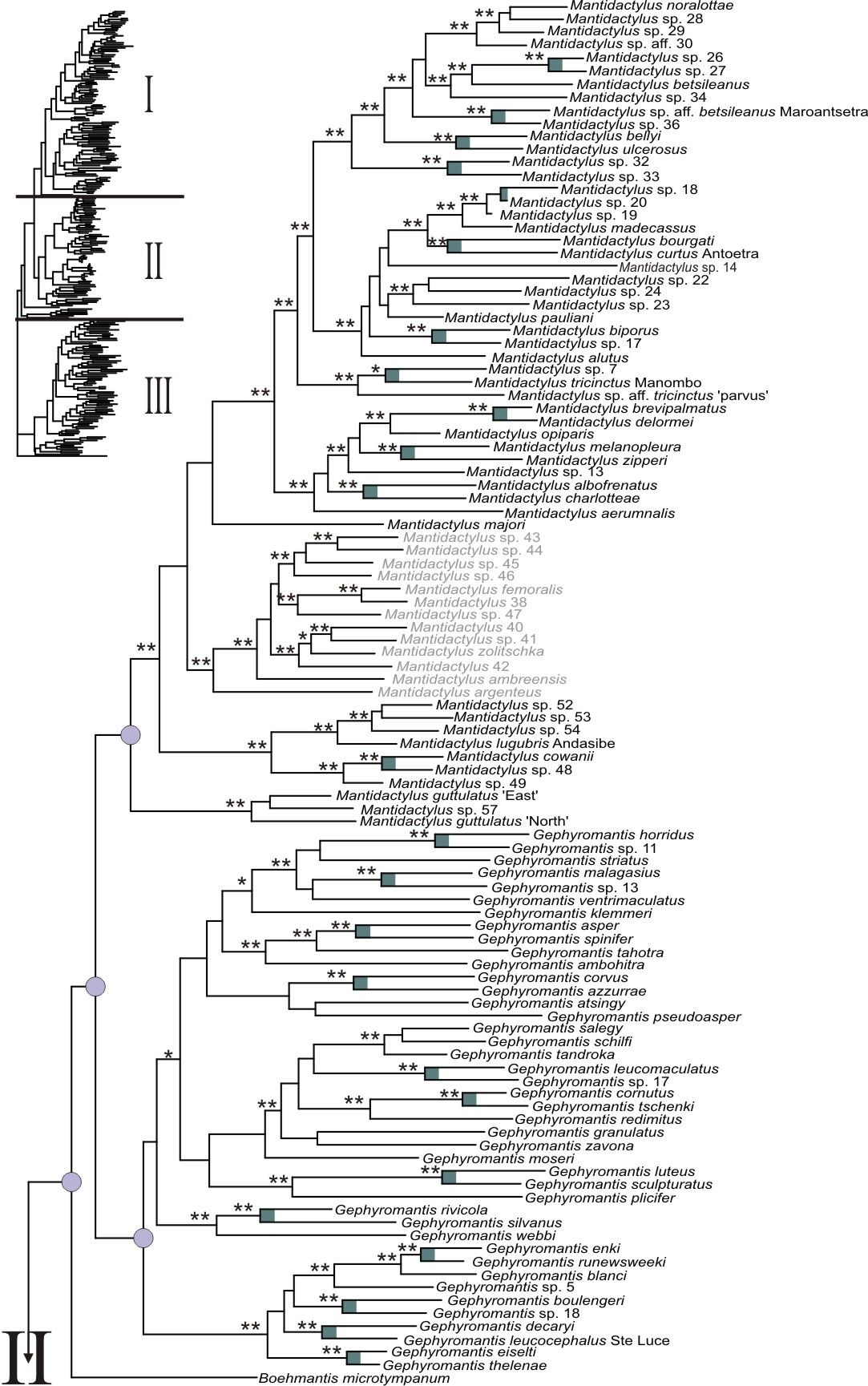


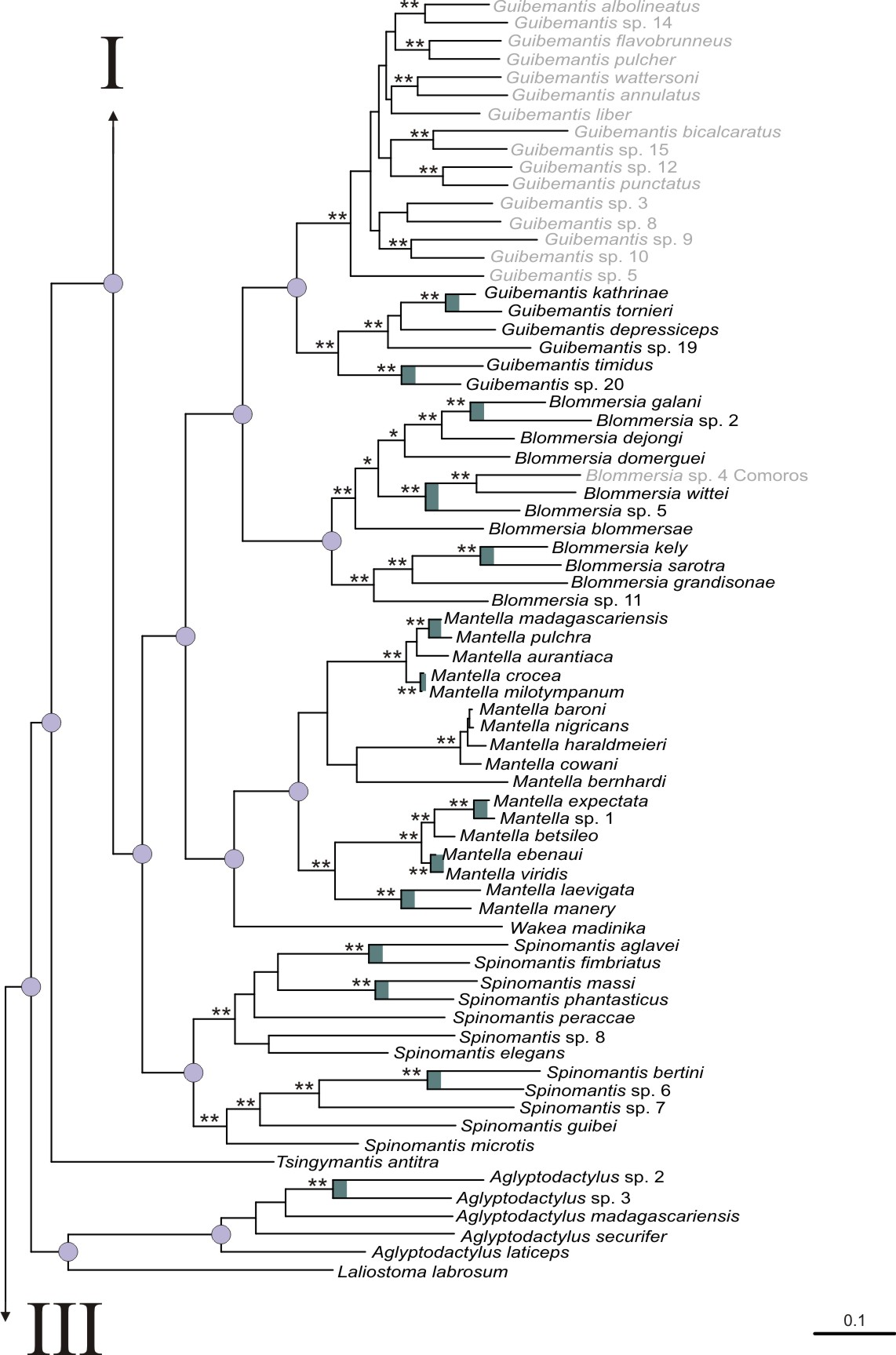


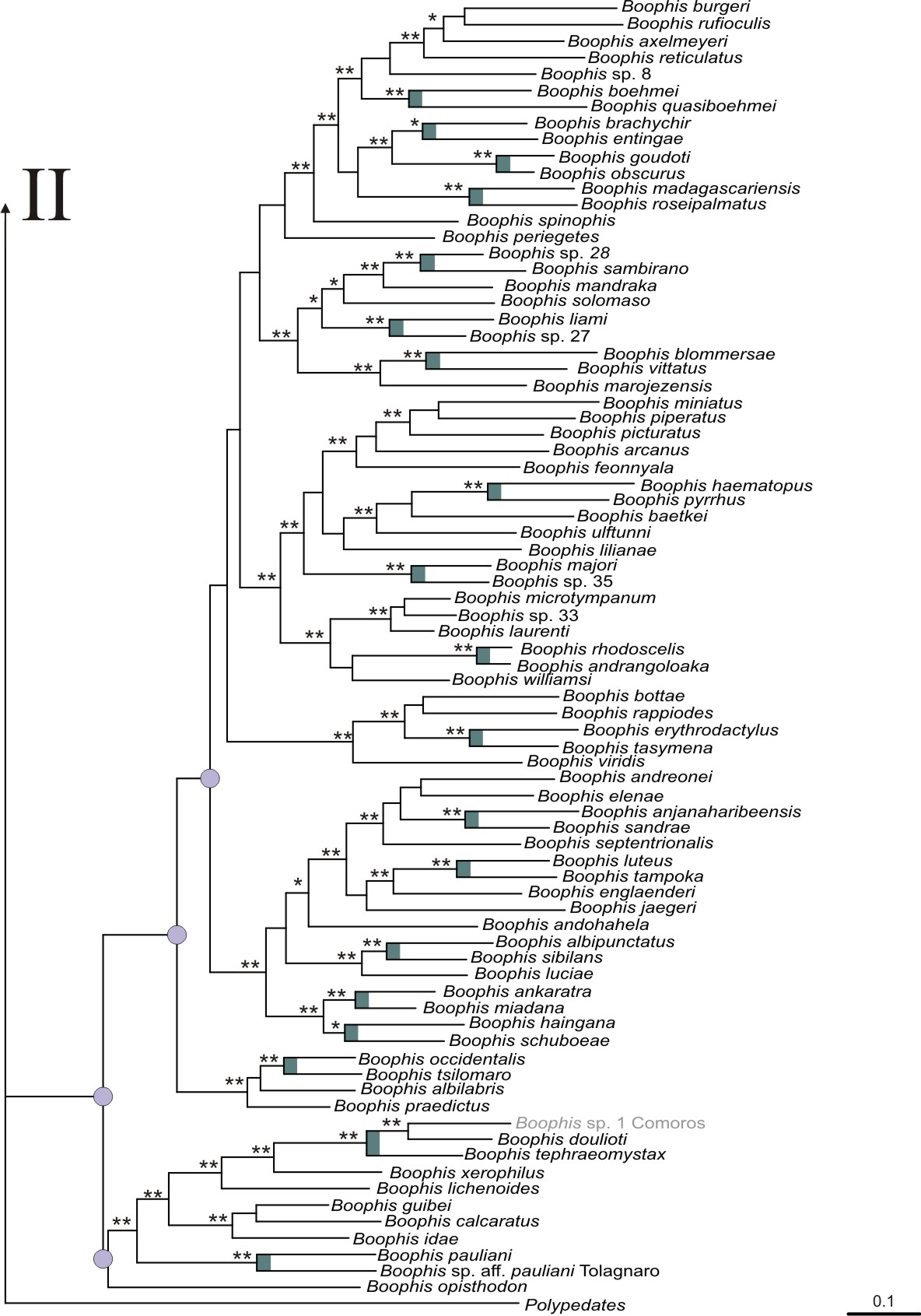


Supplementary Figure S3. Histograms for SVL, RSA, RSB and their logarithms.


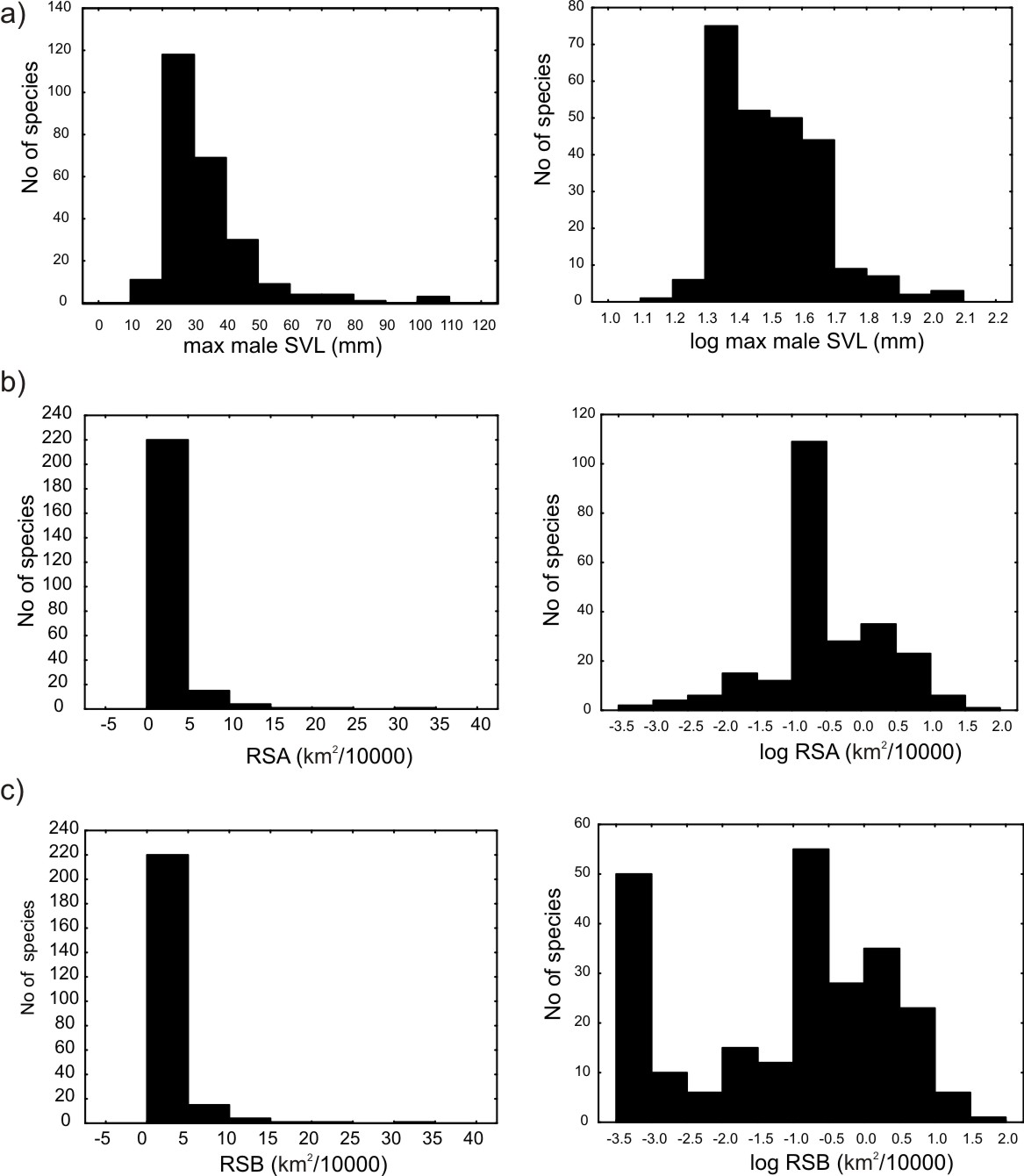

Supplement: Additional file 1 — Includes a full list of voucher specimens, Genbank accession numbers, primer sequences, a phylogenetic tree obtained on the basis of the reduced-taxa data set, as well as additional tables with more detailed results of several statistical analyses. [file 1471-2148-11-217-S1.DOC]
